# Supplementary material for: Natural Scales in Geographical Patterns
Source: Sci Rep. 2017 Apr 4;7:45823. doi: 10.1038/srep45823 (PMC5379183; doi:10.1038/srep45823)
Supplement: Supplementary Information [file srep45823-s1.pdf]

Supplementary Information for
"Natural Scales in Geographical Patterns"

Telmo Menezes and Camille Roth

Contents

1 Dataset Details **2**

1.1 Basic facts and protocol flowchart 2

1.2 Raw distance distributions 4

2 Percentiles and distance spread: low-level mobility vs. high-level patterns **5**

3 User Activity **7**

4 Maps **10**

1 Dataset Details

1.1 Basic facts and protocol flowchart

The data we retrieved from Instagram concerns user activity during a period of about 15 months, from July 1st, 2013 to October 10, 2014, using the process already described in the Materials and Methods.

Figure S1 summarises the complete workflow that we used. Data retrieval and cleaning tasks are shown in pink, operations in graph space are shown in blue and operations in Voronoi space in green. Notice that from the "community detection" step on, every operation is performed once for each distance percentile, as described in the main text.

In table S1 we present some basic metrics about the dataset for each region. *Active users* are defined as the users who contribute to movement graphs. That is to say, these are the users that took photos in at least two different locations.

Region	#users	#active users	#photos	#locations
Belgium	4 434 888	165 216	4 922 314	543
Benelux	9 625 559	376 832	11 108 872	2 905
Berlin	2 059 604	103 954	1 025 526	9 801
Israel	3 470 146	194 089	6 556 850	157
Paris	2 530 314	119 448	1 011 423	1 521
Poland	4 334 065	251 653	5 783 332	20 683
Portugal	4 151 681	185 236	5 422 643	589
Romania	1 389 381	58 176	1 928 514	3 772
Ukraine	5 260 829	307 054	15 744 788	2 098

Table S1: Some metrics about the dataset for each region.

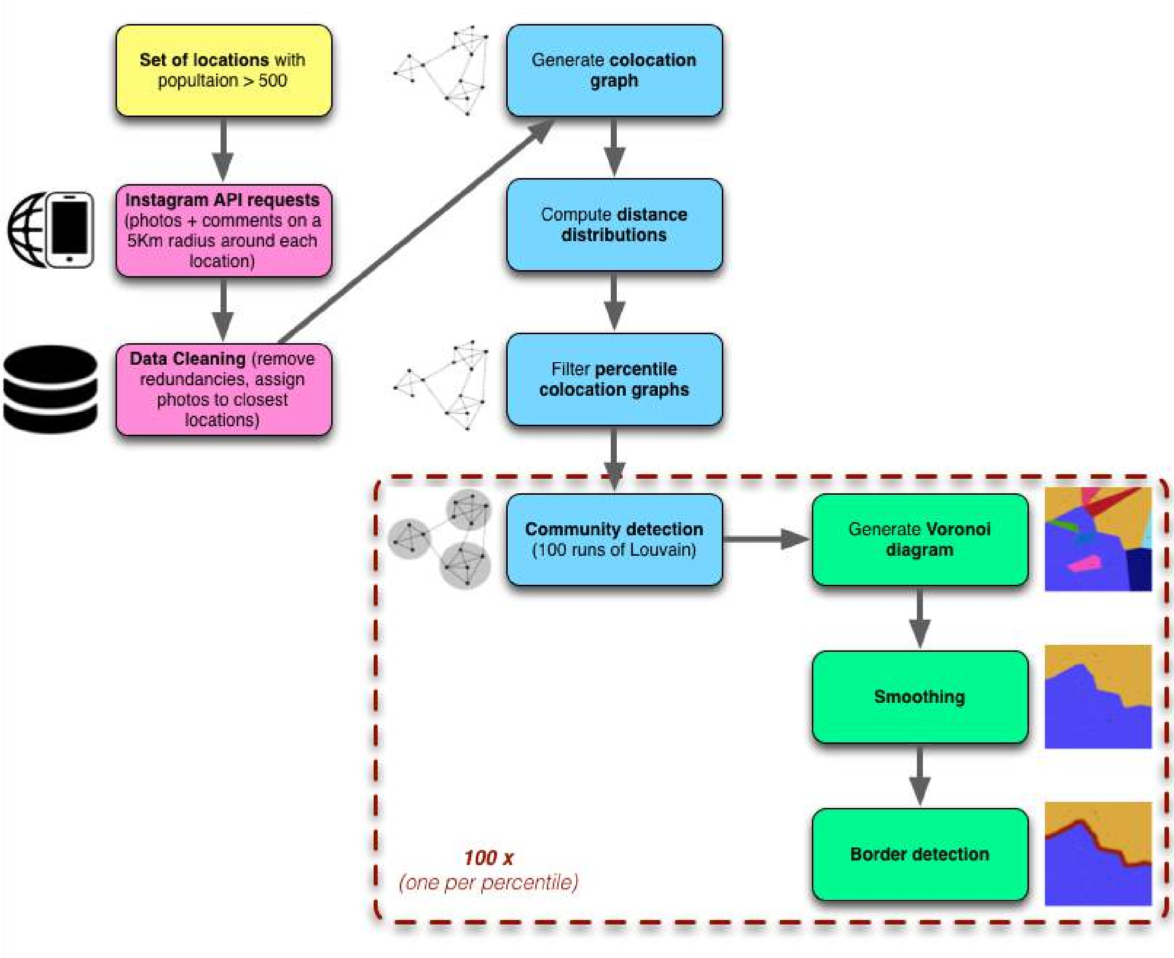

Figure S1: Protocol flowchart.

1.2 Raw distance distributions

In figure S2 we show the distance distributions for the full graph of movement between locations using log-log scales. Natural scale discontinuities (presented in the main work in terms of percentiles) can be seen here in absolute distance space (in kilometers). They are represented by the vertical red dotted lines.

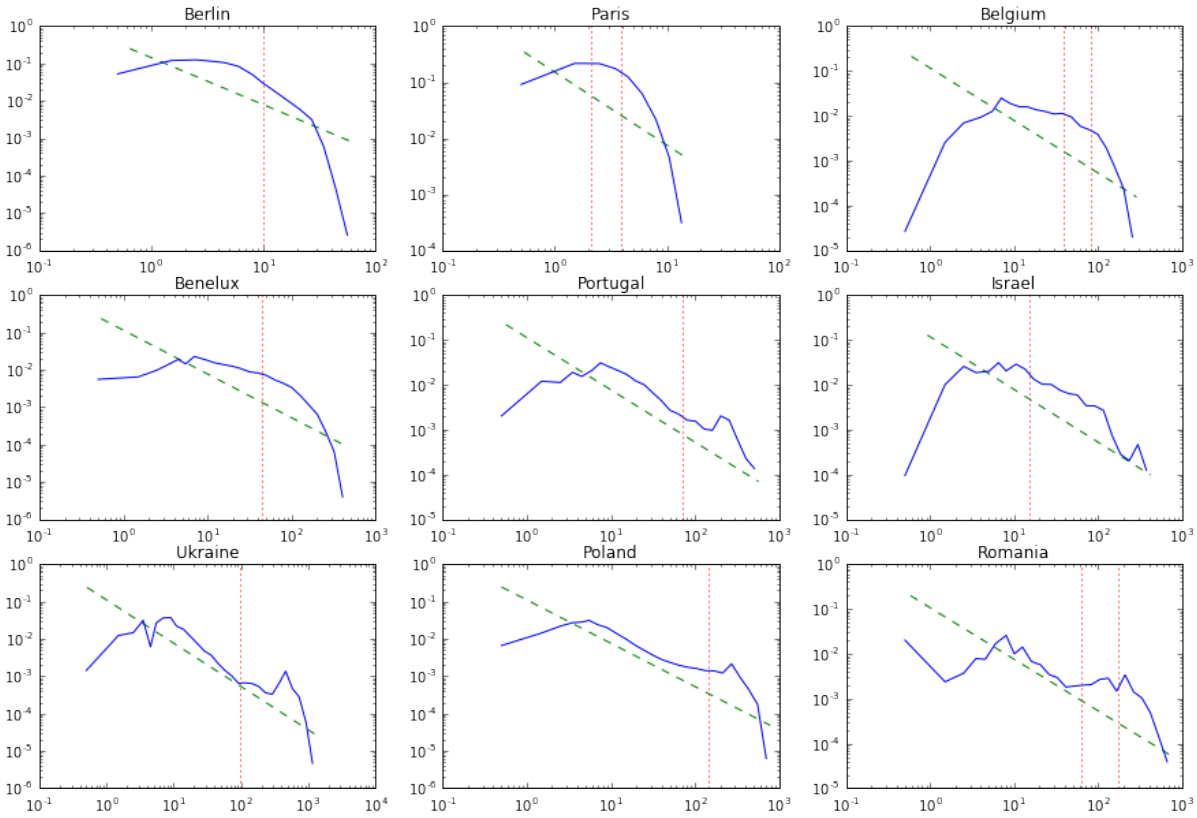

Figure S2: Distance distributions for all regions, log-log scale (x-axis: kms, y-axis: frequency). Power law fitting lines are shown in green (dashed), scale discontinuities in red (dotted).

2 Percentiles and distance spread: low-level mobility vs. high-level patterns

Percentiles are based on the ranking of edges at a certain physical distances in increasing order. As said in the main text, this yields percentile scale where scale s corresponds to a typical physical distance.

We analyse further the relation between physical distances and percentile scales. For any percentile scale s we define ρ as the typical physical distance of s relative to the median percentile. For a given region, $\rho(s)$ tells us whether mobility at percentile scale s diverges from the median physical distance of that region. Figure S3 shows the distribution of ρ for each region.

On average, the spread of physical distances on percentile scales is a rough indicator of whether a region tends to exhibit a strong heterogeneity in the range of mobility behaviour between the lowest and highest scales. The more ρ diverges from 1, the stronger this heterogeneity is. In turn, we can compute the geometric average of ρ (since ρ is a ratio) restricted to the left-side or the right-side of the distribution (where ρ is by definition respectively smaller or greater than 1), in order to know whether the heterogeneity is more marked for lower or higher scales. We thus define $\langle\rho^-\rangle$ and $\langle\rho^+\rangle$, respectively. The values for these quantities are depicted on figure S3, along with the median physical distances m. In Romania, for instance, most of the lower scales operate at markedly small physical distances (relatively to the median), while higher scales are more similar amongst each other.

A stronger concentration of mobility ranges in one or both sides of these distributions could be related to the presence of a higher or smaller proportion of small-/large-scale patterns. To assess this, we define κ as the ratio between the number of geographical patterns at the highest natural scale and the lower natural scale. In other words, κ should be lower for regions where a patchy pattern is visible for areas defined at the lowest natural scale.

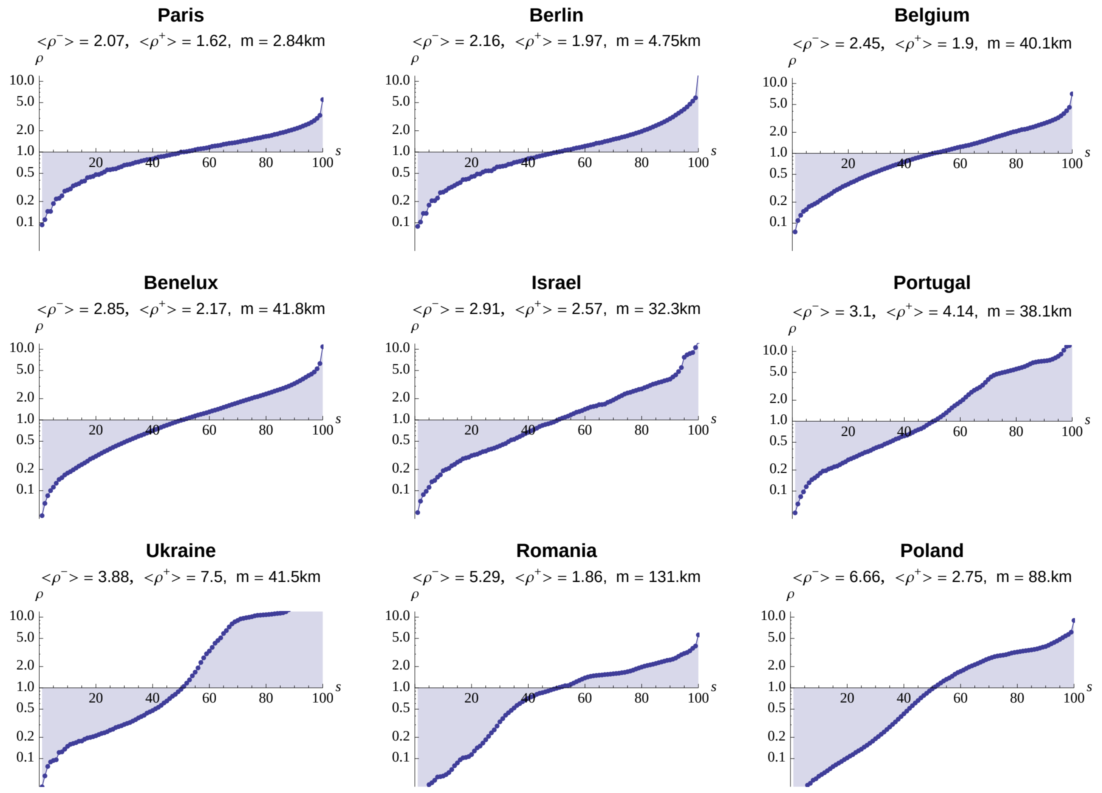

Figure S3: Typical physical distance $\rho(s)$ of links at percentile scale s relative to the median physical distance m at percentile $s = 50\%$, for each region. $\langle \rho^- \rangle$ and $\langle \rho^+ \rangle$ are geometric averages of ρ respectively for the left-side and right-side of the distributions.

Figure S4 shows that a linear regression on κ and ρ^- yields a relatively good fit ($R^2 = 0.70$), suggesting that there is typically a direct relationship between the patchy character of geographical patterns at the lowest natural scale and the spread of the smallest physical range for user mobility. Visual examination of figure 3 confirms that regions to the right of this diagram exhibit the highest proportion of small, lower-scale regions.

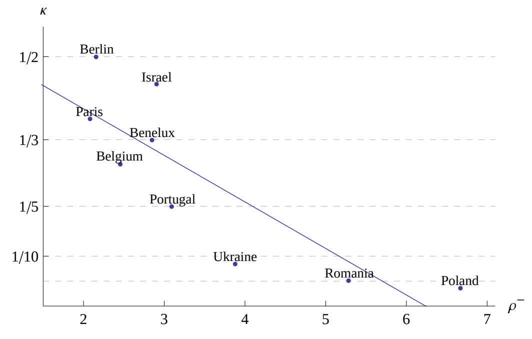

Figure S4: Relationship between κ and ρ^-. The solid line represents the best linear fit $\kappa = -0.10\rho^- + \text{constant}$, achieving a R^2 of 0.70.

3 User Activity

To have a better understanding on the distribution of the level of user activity, we plotted the charts shown in figure S5 and figure S6. The former shows the distribution of number of photos taken by user, the latter the number of different locations where a user was active.

Figure S7 complements the figure 4 in the main text by depicting scale-specific slices using the number of photos instead of the number of locations, showing that core users contributing to all scales are not only the most active geographically (in terms of visited locations) but also in terms of pure photo activity (number of posted photos).

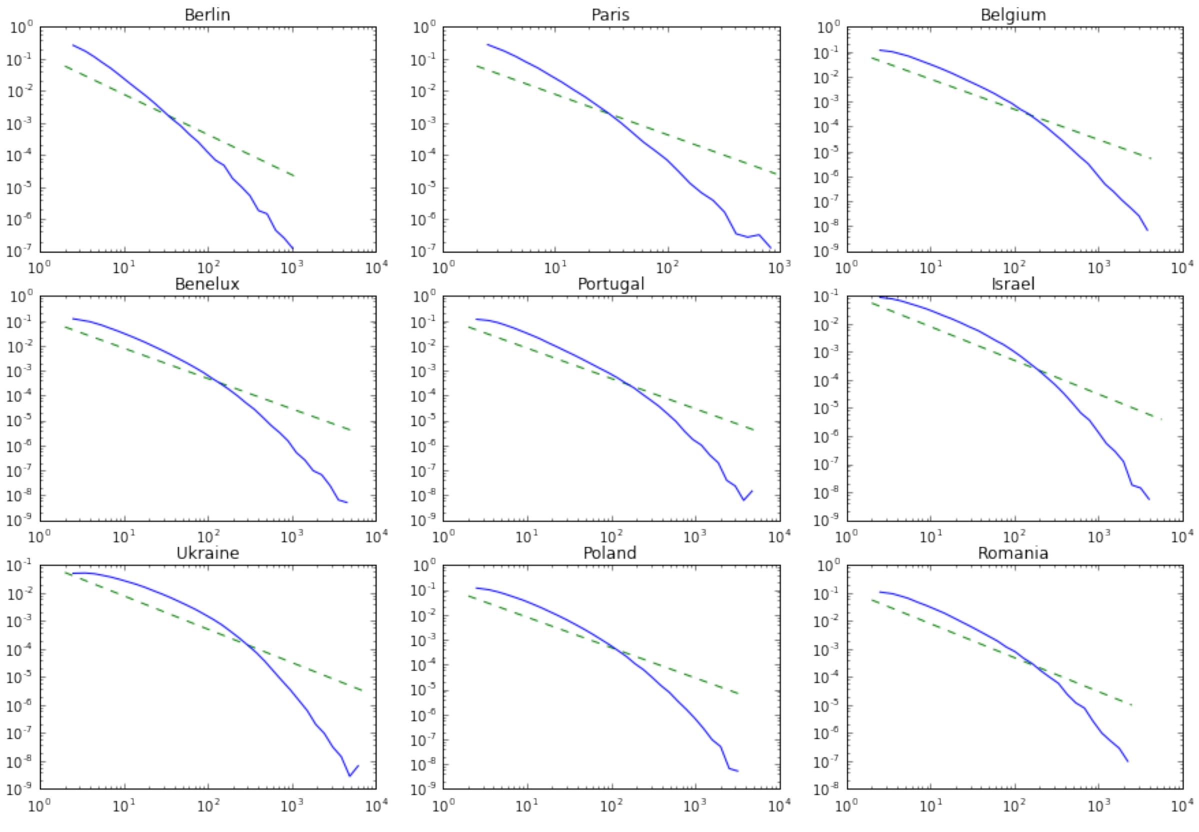

Figure S5: Photos per user, log-log scale (x-axis: photos, y-axis: frequency). Power law fitting lines are shown in green (dashed).

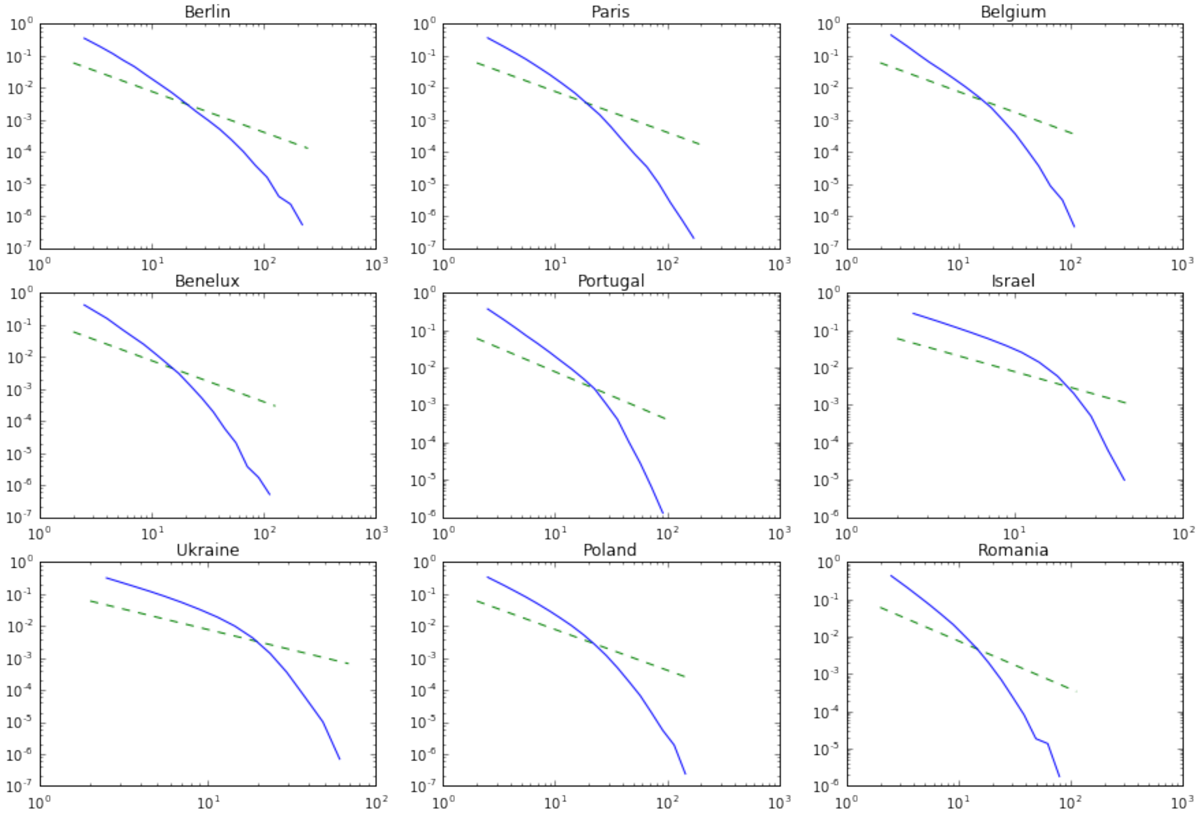

Figure S6: Locations per user, log-log scale (x-axis: locations, y-axis: frequency). Power law fitting lines are shown in green (dashed).

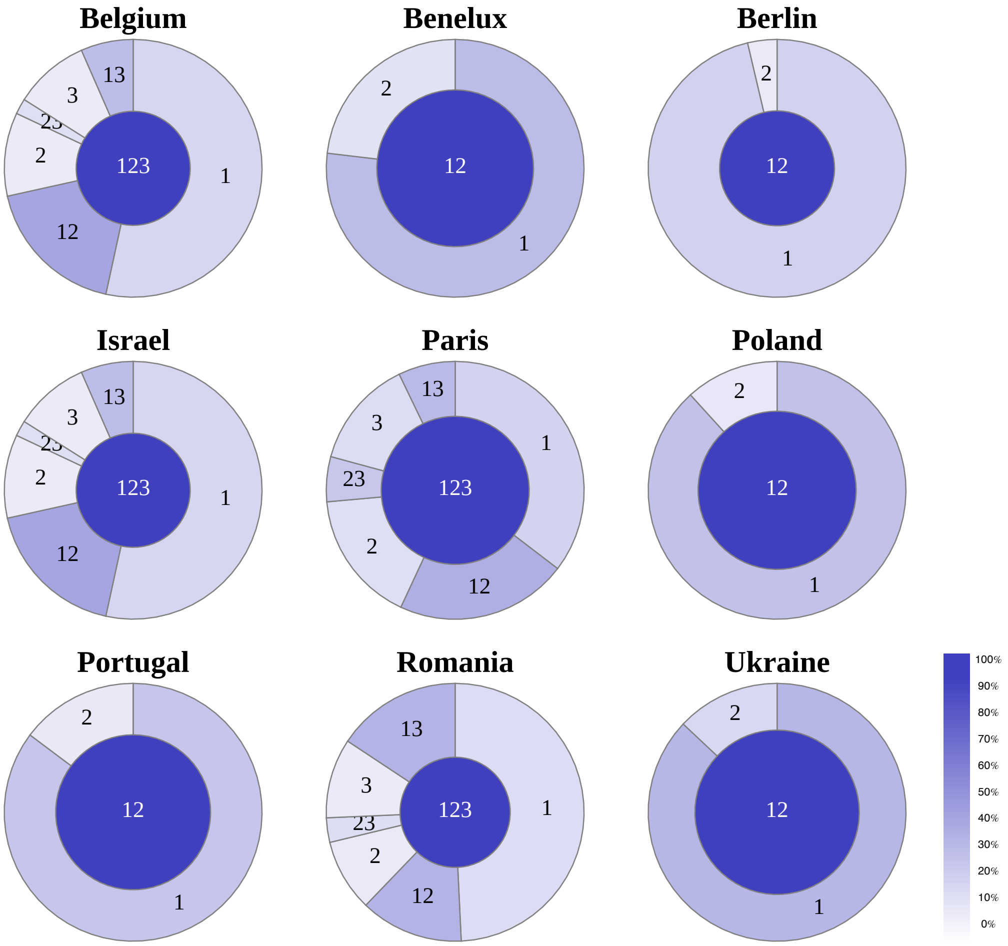

Figure S7: Charts representing the fraction of users contributing to each natural scale. The area of each slice/circle is proportional to the number of users active in the set of scales that it represents ("12" corresponds to users contributing exactly to scales 1 and 2). User activity is represented by slice darkness, which is proportional to the number of photos taken relative to the maximal activity of a given region (100%): here, "123" users are always the darkest / most active slice, they consistently take many more photos than other users.

4 Maps

We have produced a number of detailed maps for each region under study. Due to space constraints they cannot be shown in full detail in the main text, so we present them here. For each region we show maps with geographical boundaries at each prototypical scale, as well as the multi-scale synthesis. Figures S8, S9, S10, S11 are for Belgium; S12, S13, S14 for Benelux; S15, S16, S17 for Berlin; S18, S19, S20 for Israel; S21, S22, S23, S24 for Paris; S25, S26, S27 for Poland; S28, S29, S30 for Portugal; S31, S32, S33, S34 for Romania; and S35, S36, S37 for the Ukraine.

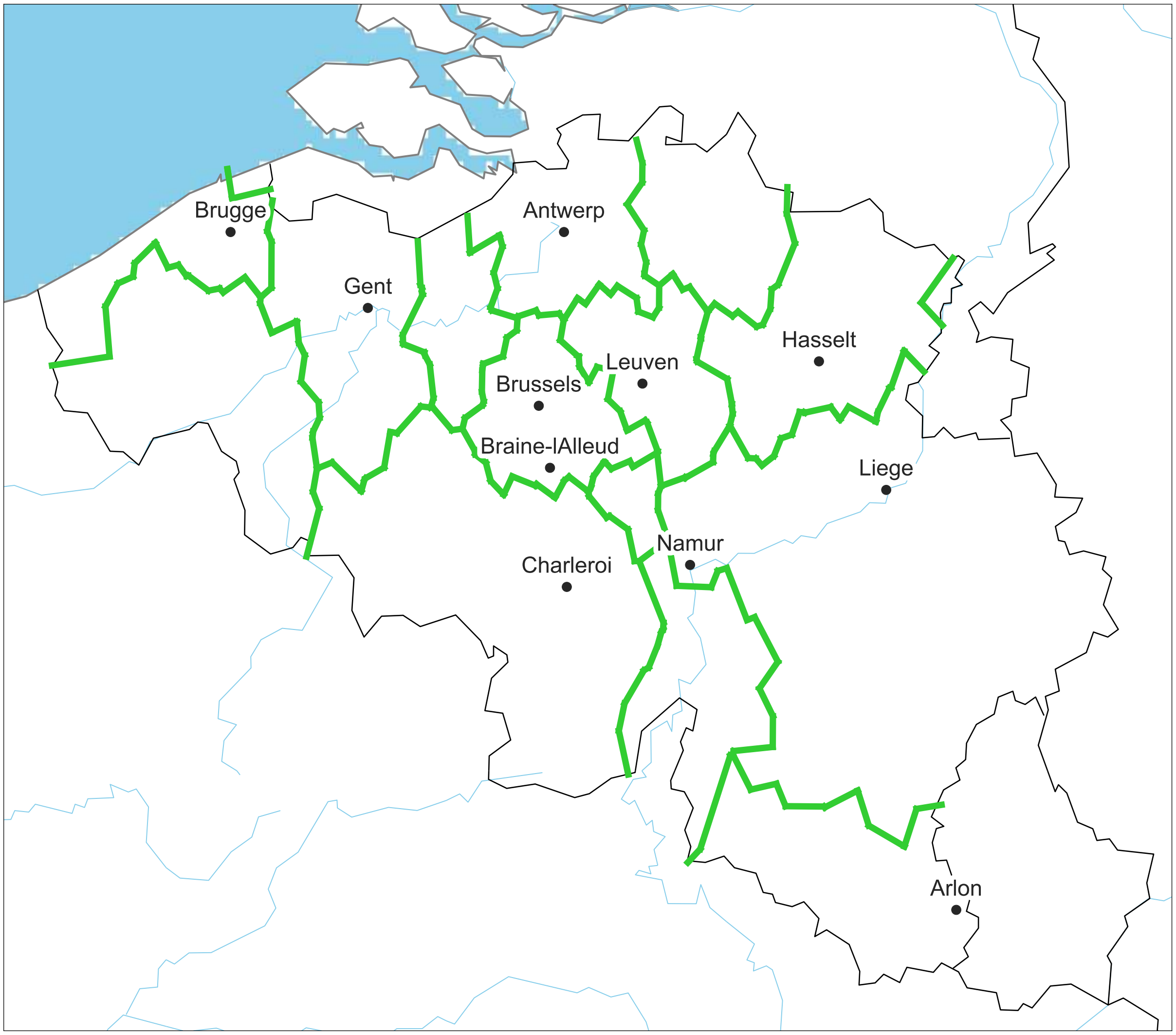

Figure S8: Belgium, short-distance borders (percentile 18). Map generated by the authors using the Basemap Matplotlib Toolkit ver. 1.0.8 (http://matplotlib.org/basemap/).

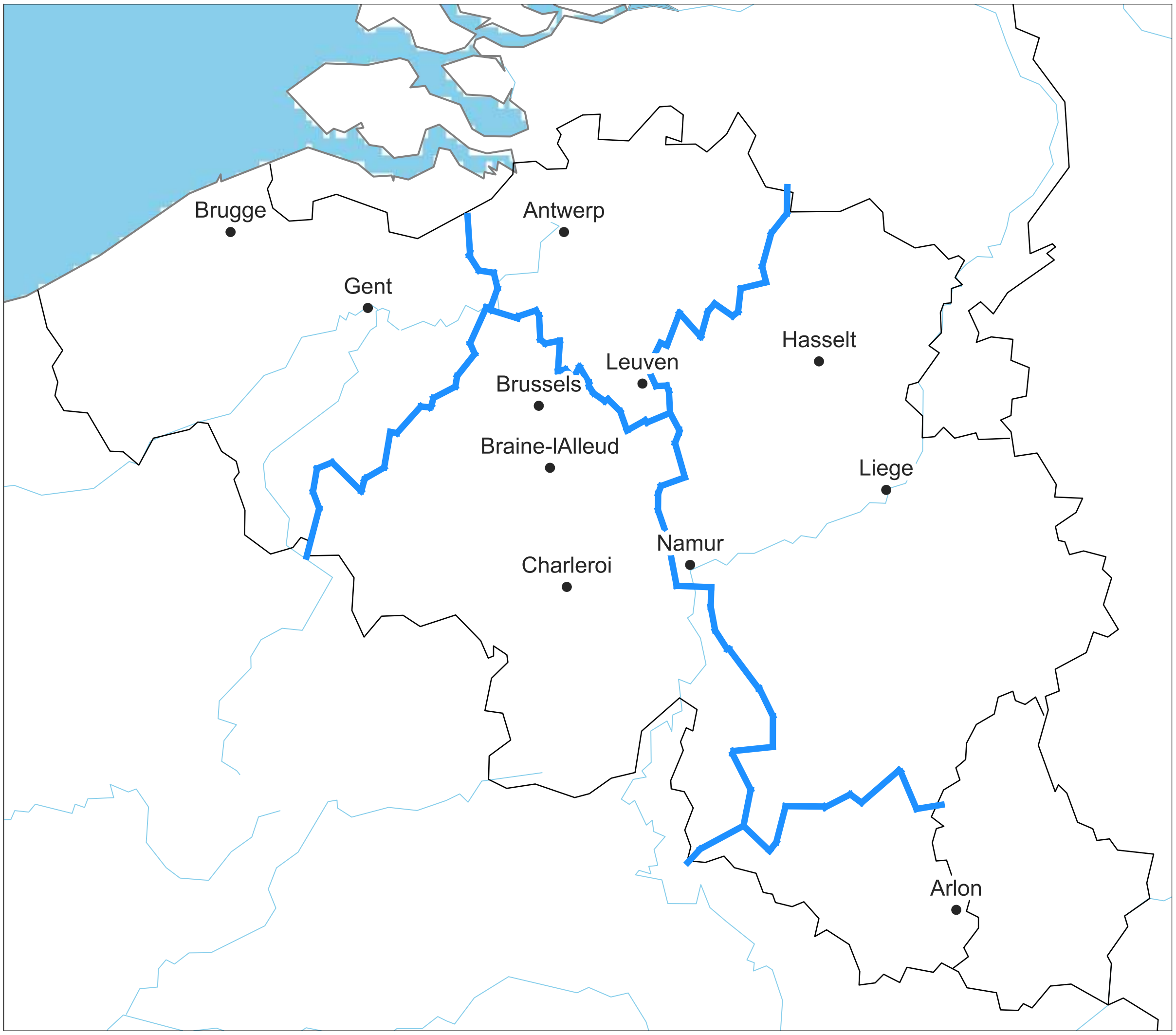

Figure S9: Belgium, medium-distance borders (percentile 65). Map generated by the authors using the Basemap Matplotlib Toolkit ver. 1.0.8 (http://matplotlib.org/basemap/).

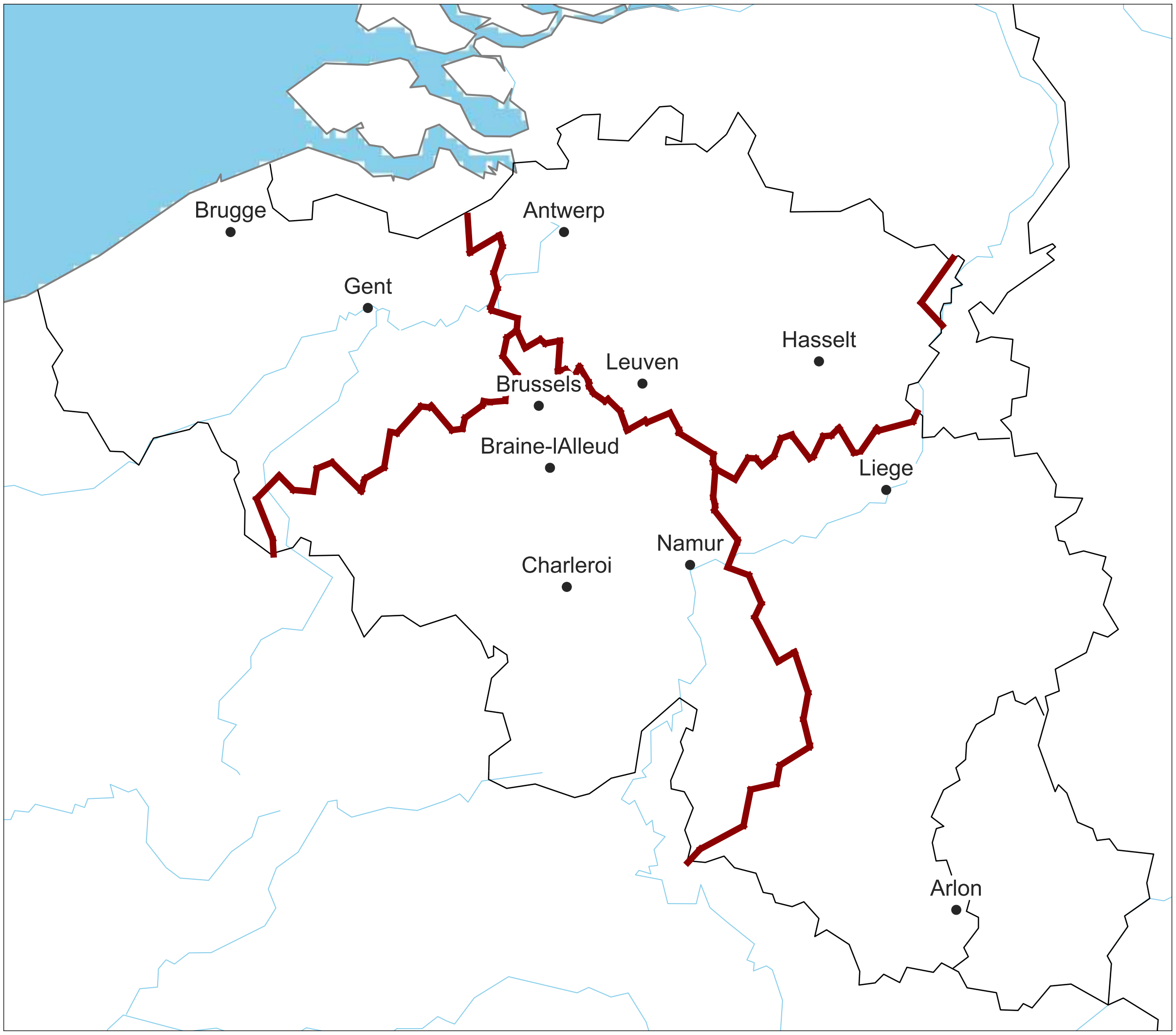

Figure S10: Belgium, long-distance borders (percentile 90). Map generated by the authors using the Basemap Matplotlib Toolkit ver. 1.0.8 (http://matplotlib.org/basemap/).

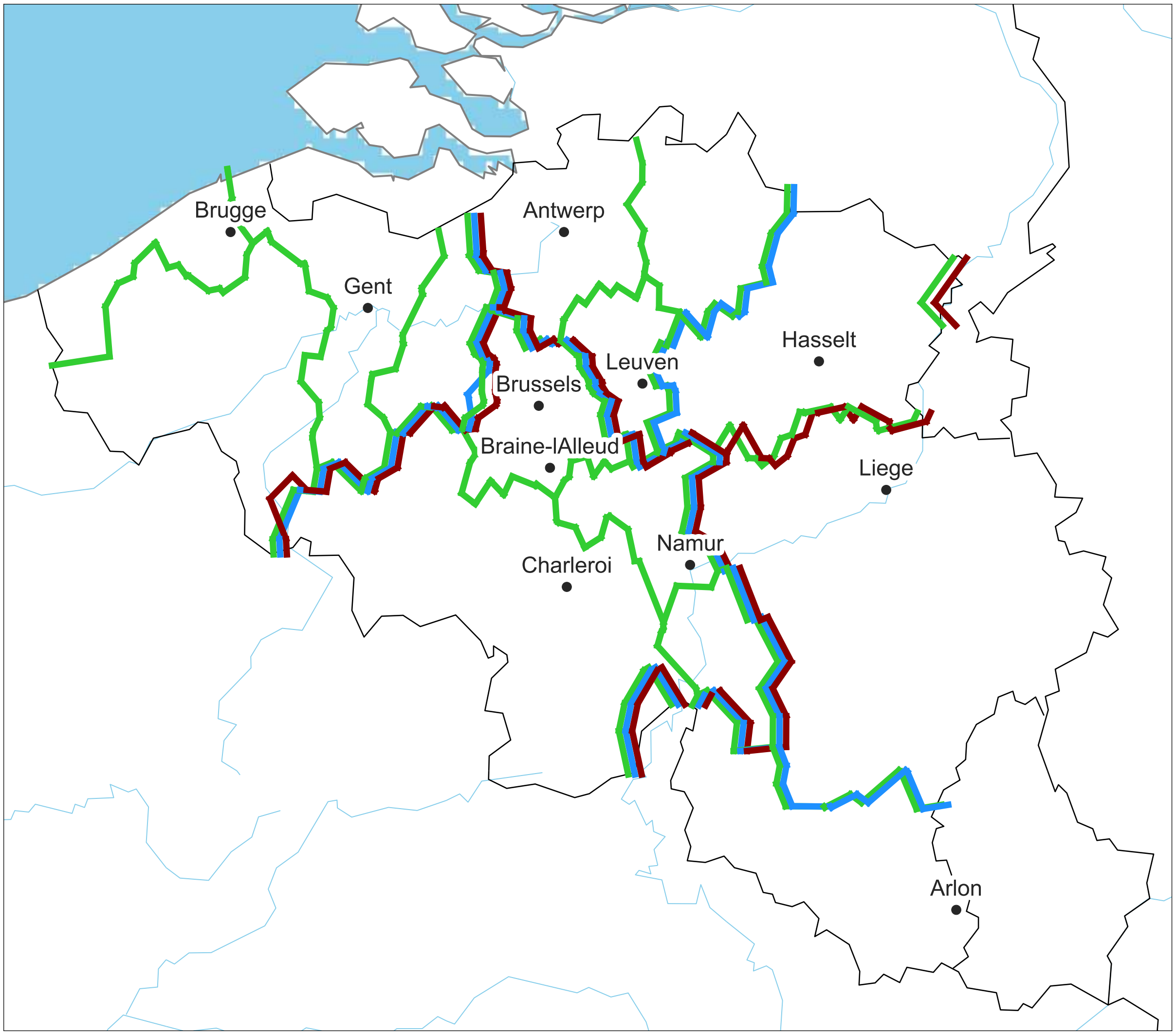

Figure S11: Belgium, multi-scale borders. Map generated by the authors using the Basemap Matplotlib Toolkit ver. 1.0.8 (http://matplotlib.org/basemap/).

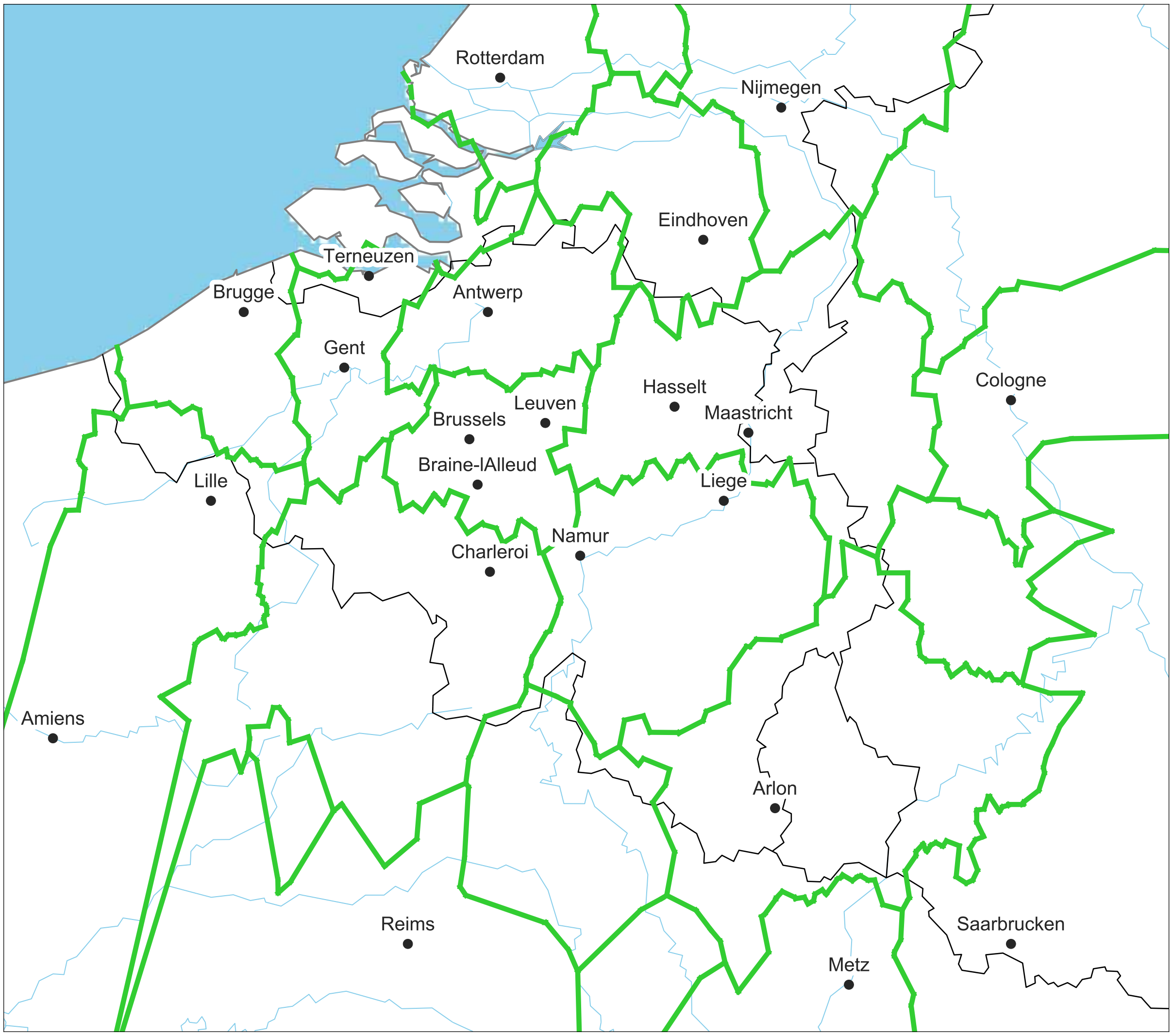

Figure S12: Benelux, short-distance borders (percentile 25). Map generated by the authors using the Basemap Matplotlib Toolkit ver. 1.0.8 (http://matplotlib.org/basemap/).

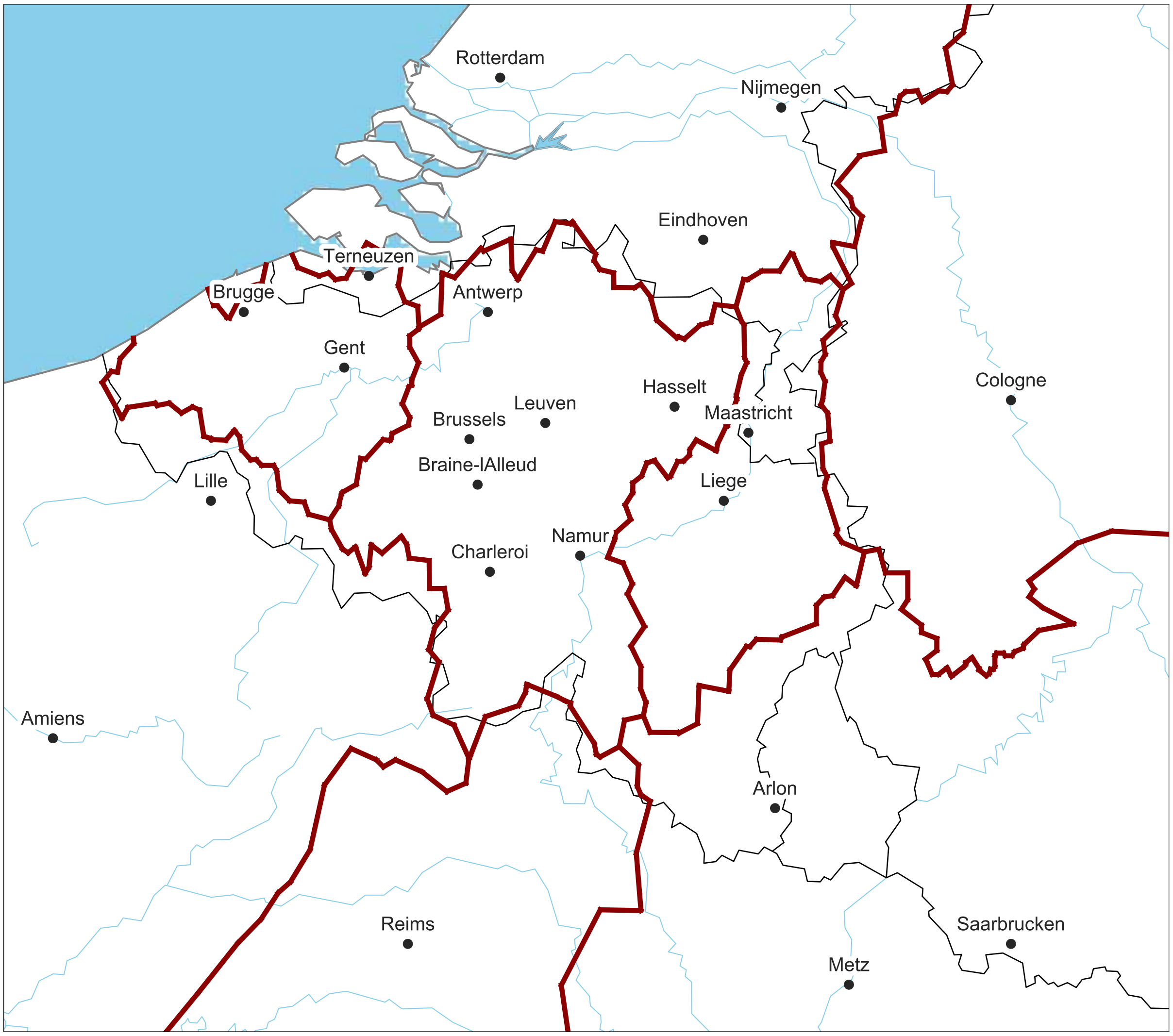

Figure S13: Benelux, long-distance borders (percentile 74). Map generated by the authors using the Basemap Matplotlib Toolkit ver. 1.0.8 (http://matplotlib.org/basemap/).

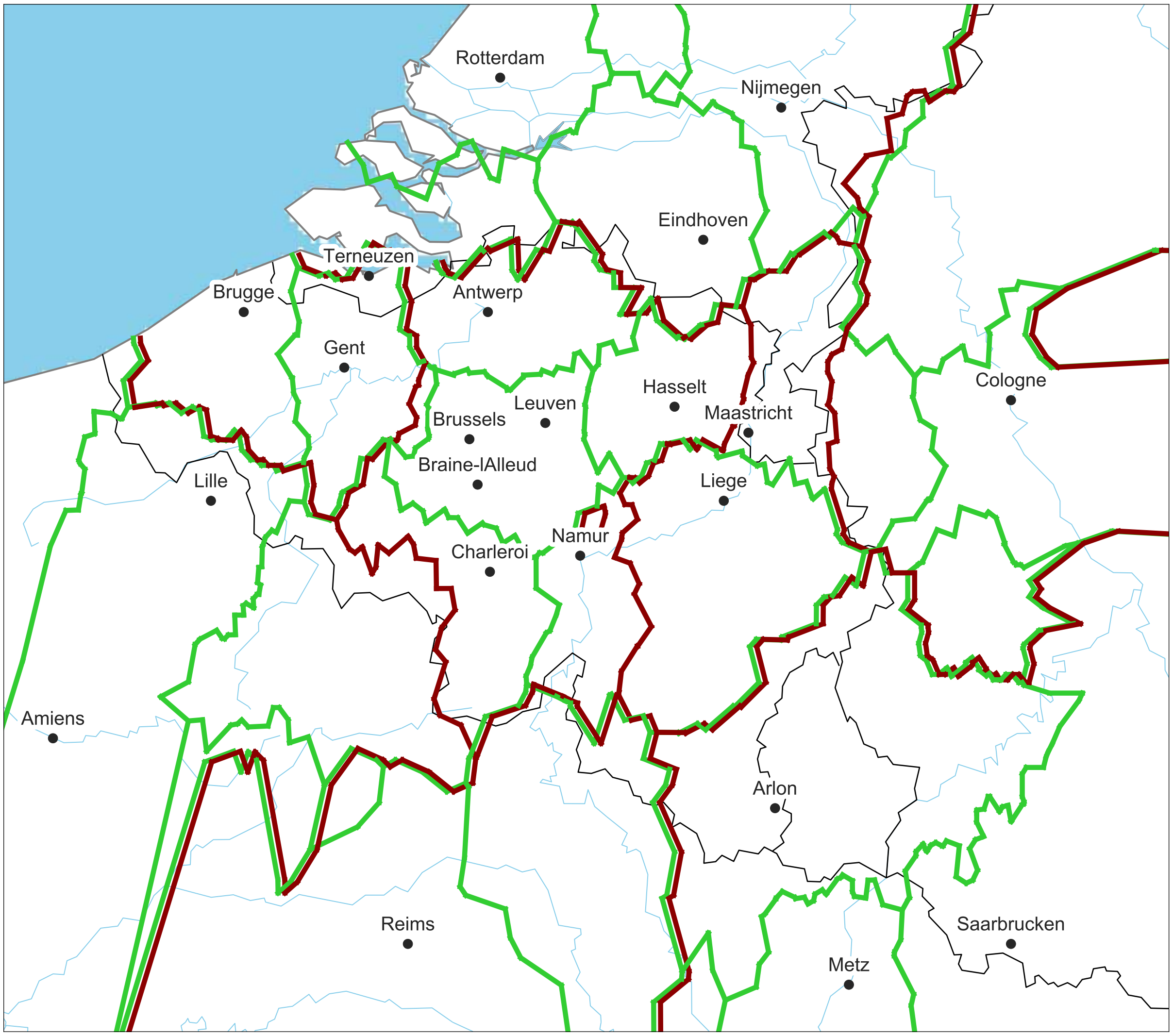

Figure S14: Benelux, multi-scale borders. Map generated by the authors using the Basemap Matplotlib Toolkit ver. 1.0.8 (http://matplotlib.org/basemap/).

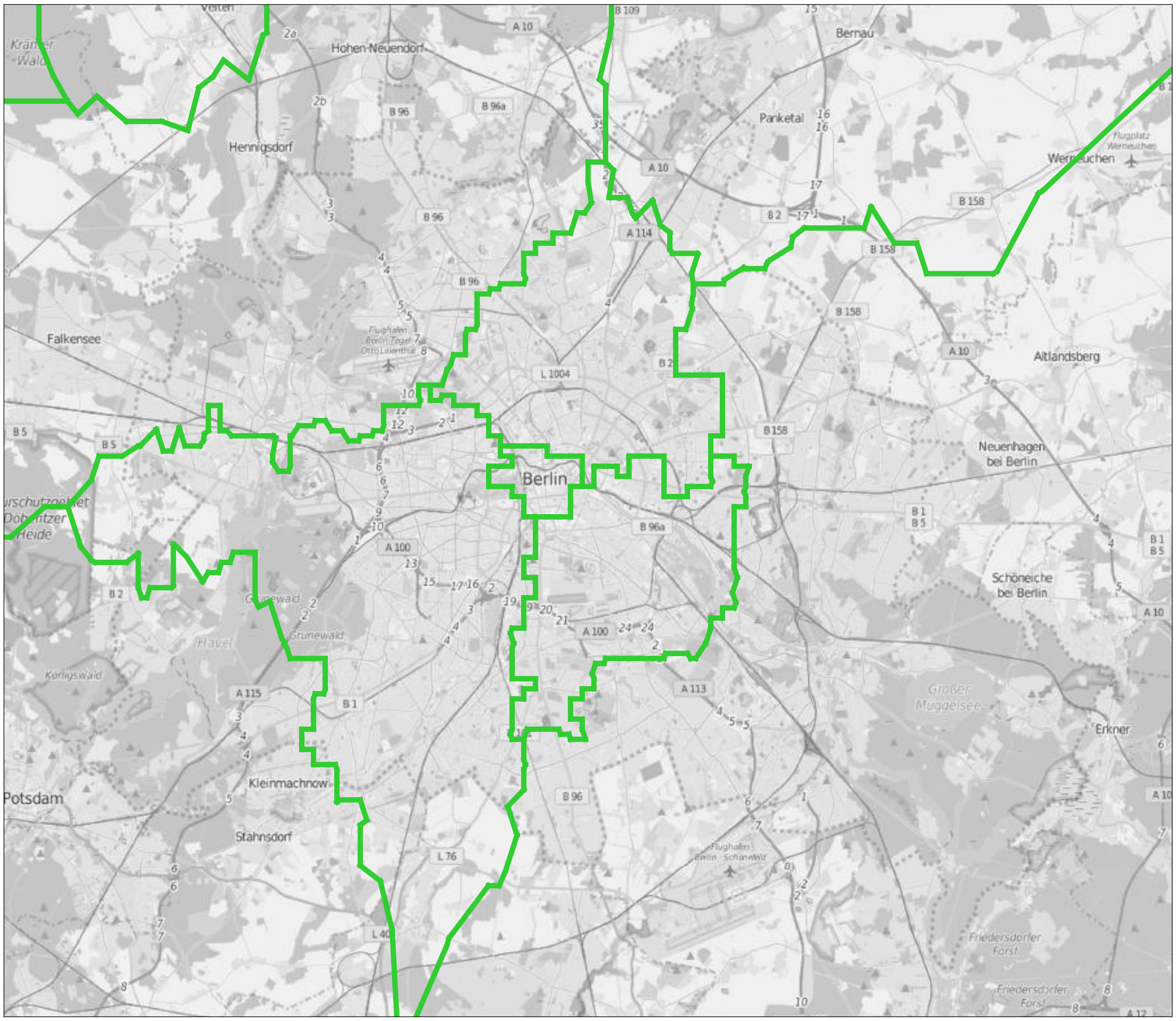

Figure S15: Berlin, short-distance borders (percentile 30). Map generated by the authors using the Basemap Matplotlib Toolkit ver. 1.0.8 (http://matplotlib.org/basemap/). Map tiles used in the background ©OpenStreetMap contributors, licensed under CC BY-SA (www.openstreetmap.org/copyright). The licence terms can be found on the following link: http://creativecommons.org/licenses/by-sa/2.0/ .

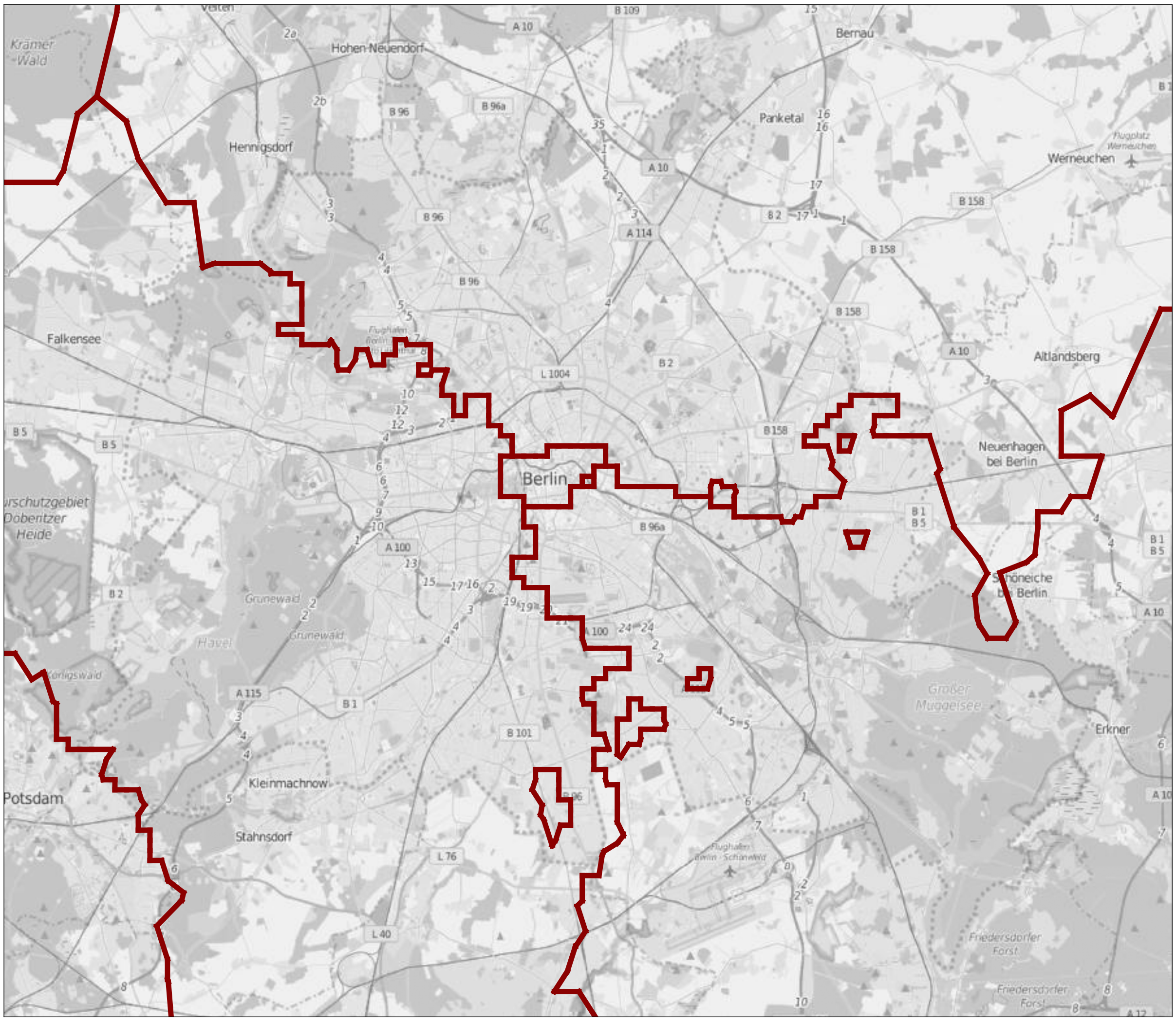

Figure S16: Berlin, long-distance borders (percentile 87). Map generated by the authors using the Basemap Matplotlib Toolkit ver. 1.0.8 (http://matplotlib.org/basemap/). Map tiles used in the background ©OpenStreetMap contributors, licensed under CC BY-SA (www.openstreetmap.org/copyright). The licence terms can be found on the following link: http://creativecommons.org/licenses/by-sa/2.0/ .

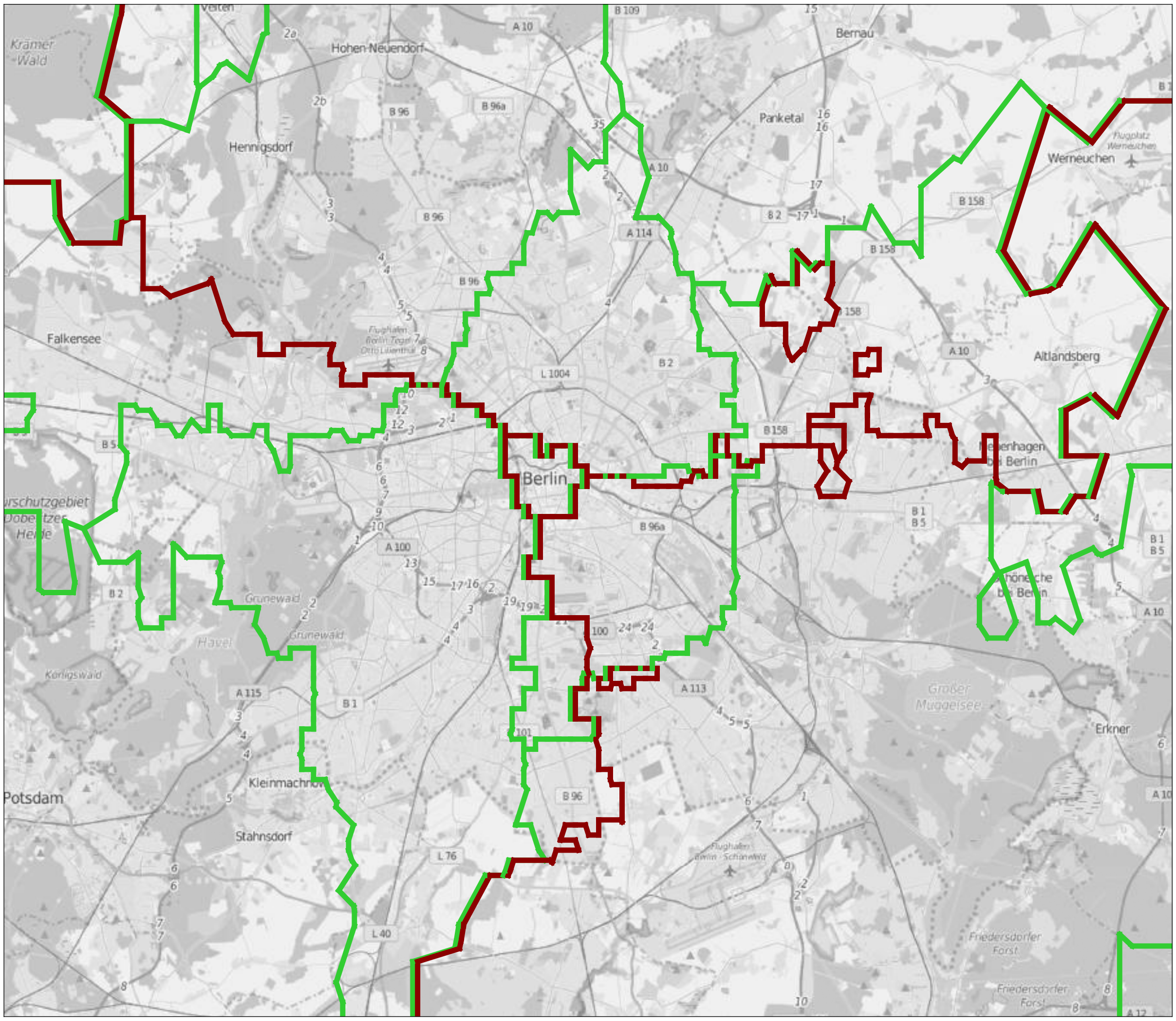

Figure S17: Berlin, multi-scale borders. Map generated by the authors using the Basemap Matplotlib Toolkit ver. 1.0.8 (http://matplotlib.org/basemap/). Map tiles used in the background ©OpenStreetMap contributors, licensed under CC BY-SA (www.openstreetmap.org/copyright). The licence terms can be found on the following link: http://creativecommons.org/licenses/by-sa/2.0/ .

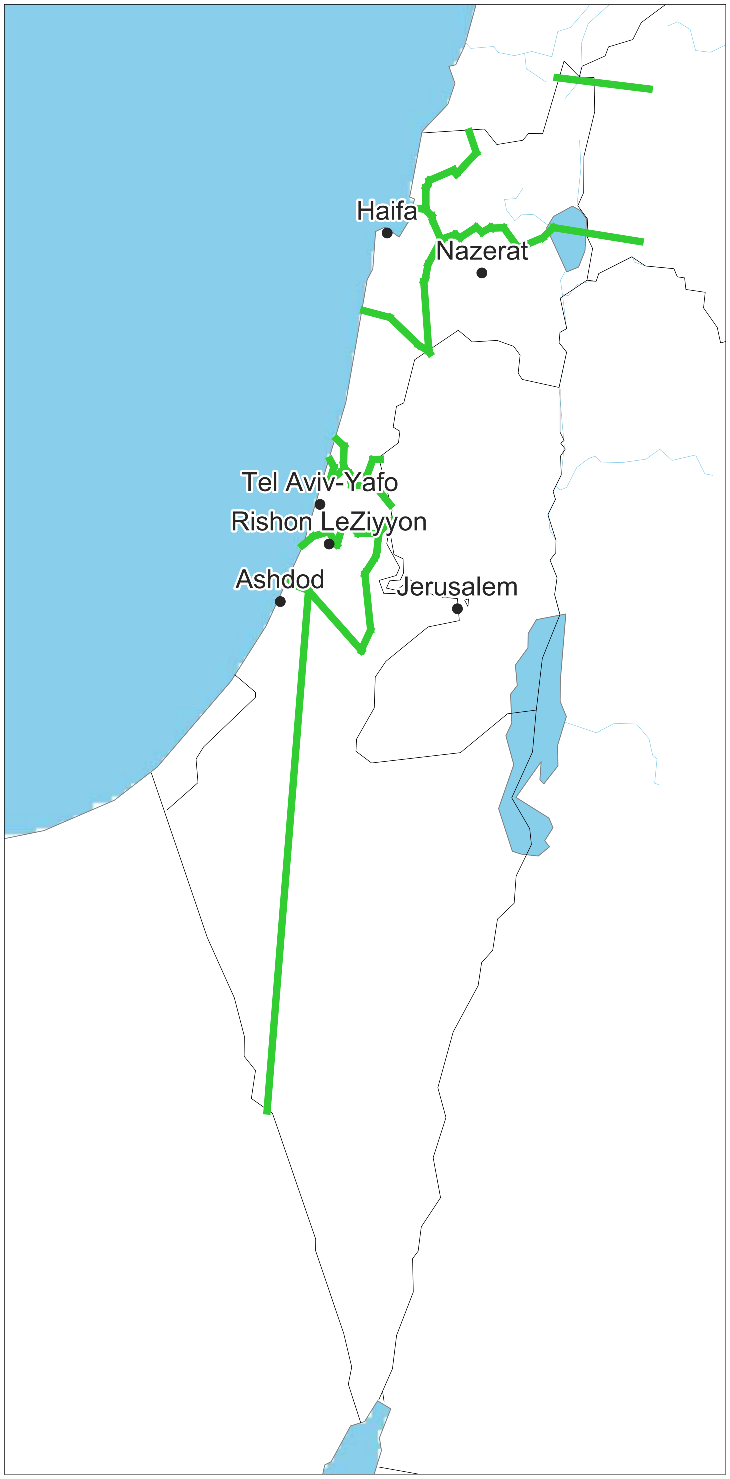

Figure S18: Israel, short-distance borders (percentile 19). Map generated by the authors using the Basemap Matplotlib Toolkit ver. 1.0.8 (http://matplotlib.org/basemap/).

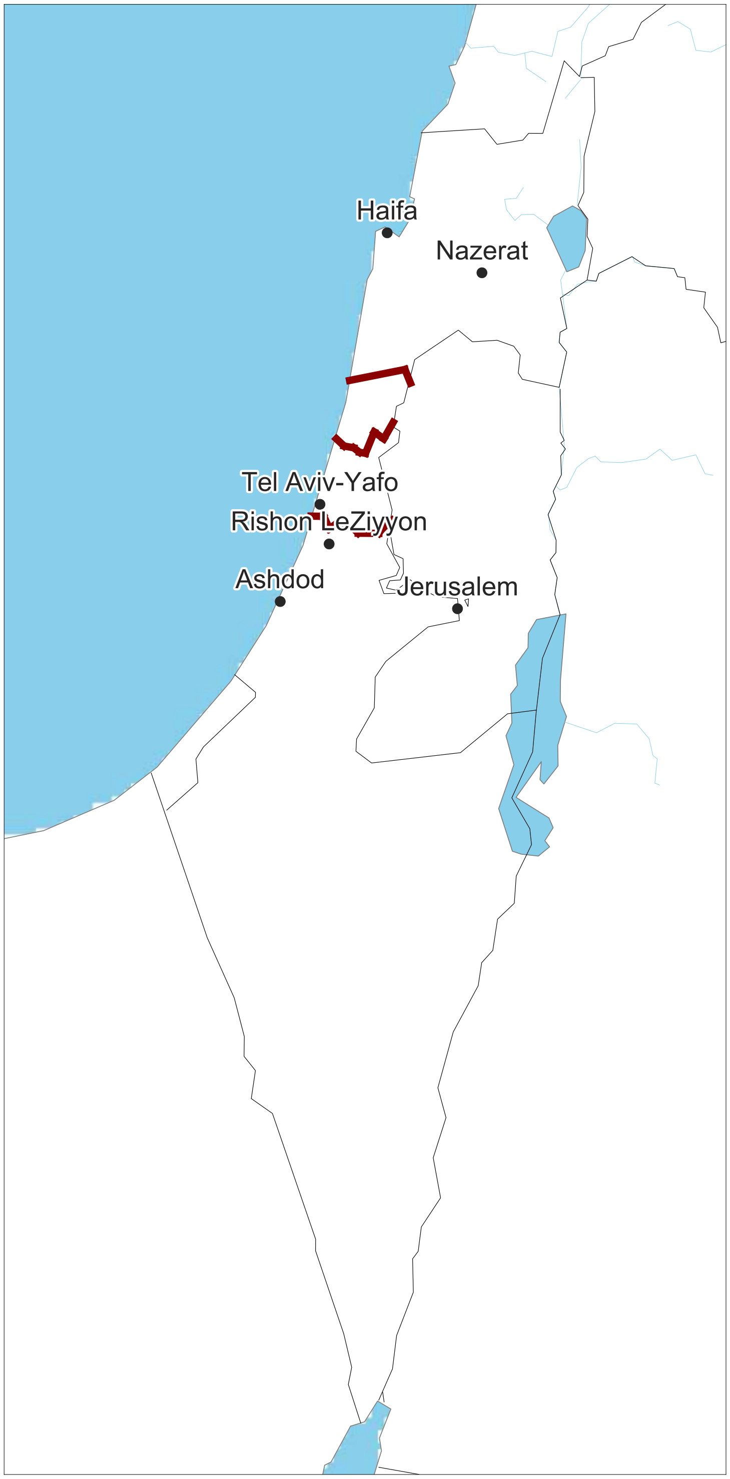

Figure S19: Israel, long-distance borders (percentile 46). Map generated by the authors using the Basemap Matplotlib Toolkit ver. 1.0.8 (http://matplotlib.org/basemap/).

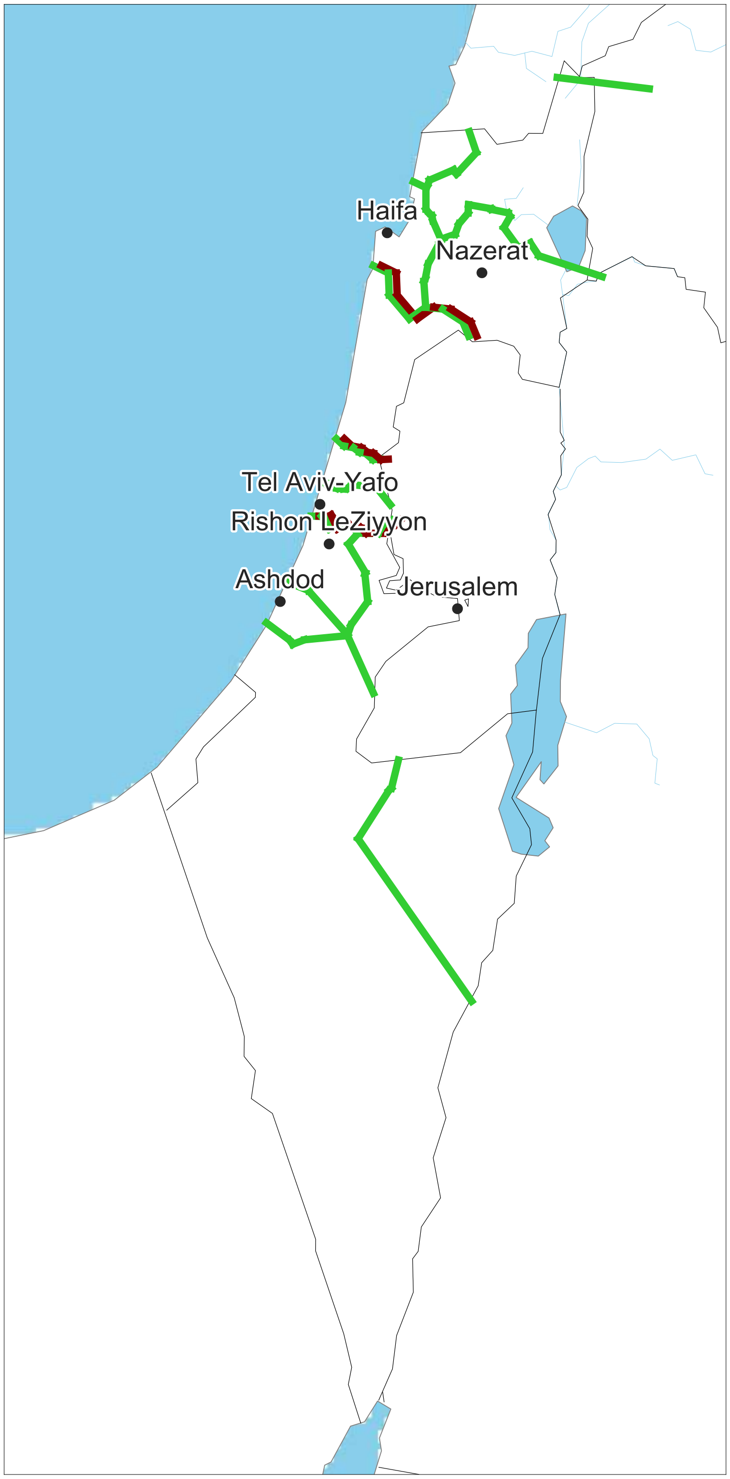

Figure S20: Israel, multi-scale borders. Map generated by the authors using the Basemap Matplotlib Toolkit ver. 1.0.8 (http://matplotlib.org/basemap/).

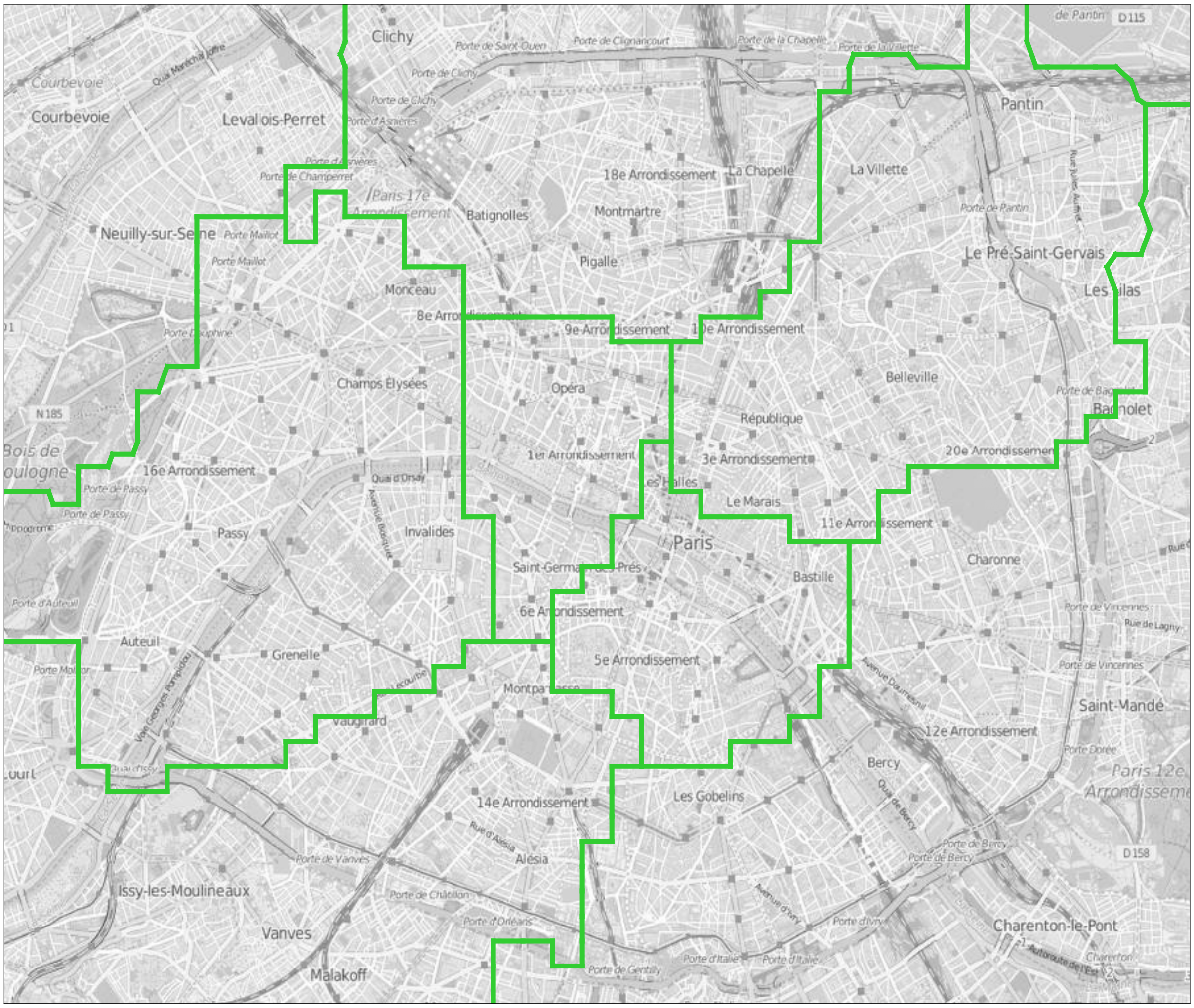

Figure S21: Paris, short-distance borders (percentile 24). Map generated by the authors using the Basemap Matplotlib Toolkit ver. 1.0.8 (http://matplotlib.org/basemap/). Map tiles used in the background ©OpenStreetMap contributors, licensed under CC BY-SA (www.openstreetmap.org/copyright). The licence terms can be found on the following link: http://creativecommons.org/licenses/by-sa/2.0/ .

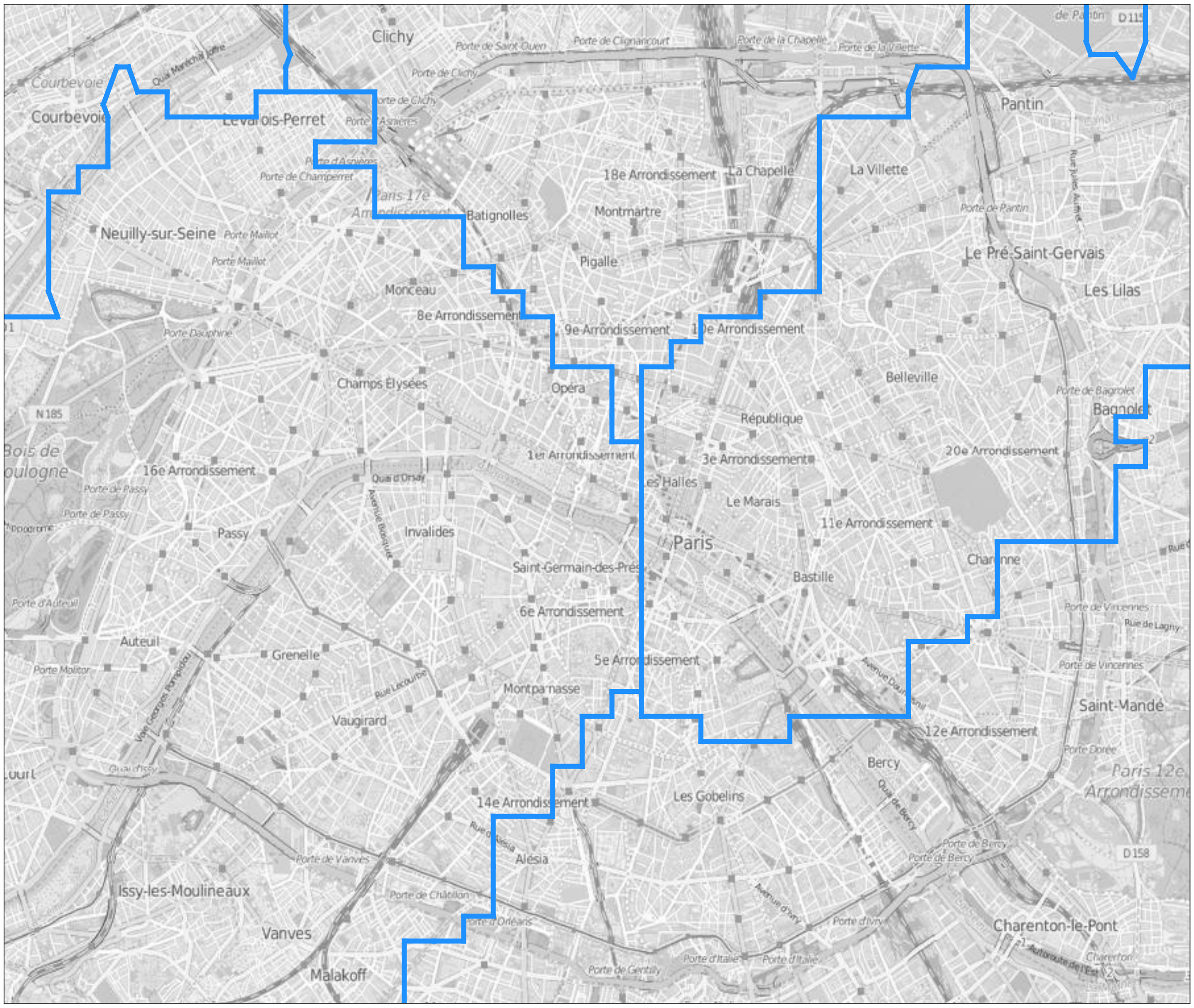

Figure S22: Paris, medium-distance borders (percentile 54). Map generated by the authors using the Basemap Matplotlib Toolkit ver. 1.0.8 (http://matplotlib.org/basemap/). Map tiles used in the background ©OpenStreetMap contributors, licensed under CC BY-SA (www.openstreetmap.org/copyright). The licence terms can be found on the following link: http://creativecommons.org/licenses/by-sa/2.0/ .

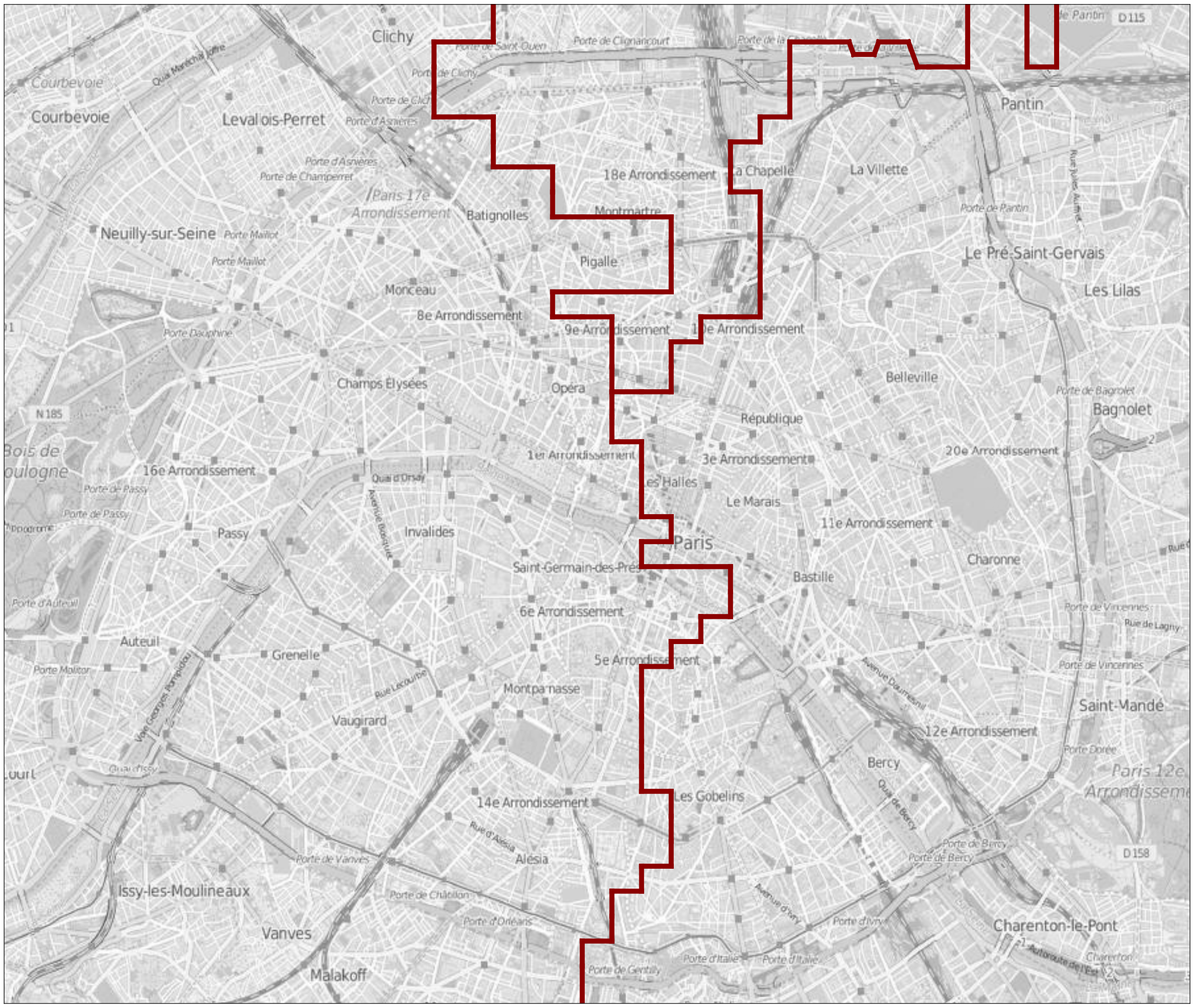

Figure S23: Paris, long-distance borders (percentile 84). Map generated by the authors using the Basemap Matplotlib Toolkit ver. 1.0.8 (http://matplotlib.org/basemap/). Map tiles used in the background ©OpenStreetMap contributors, licensed under CC BY-SA (www.openstreetmap.org/copyright). The licence terms can be found on the following link: http://creativecommons.org/licenses/by-sa/2.0/ .

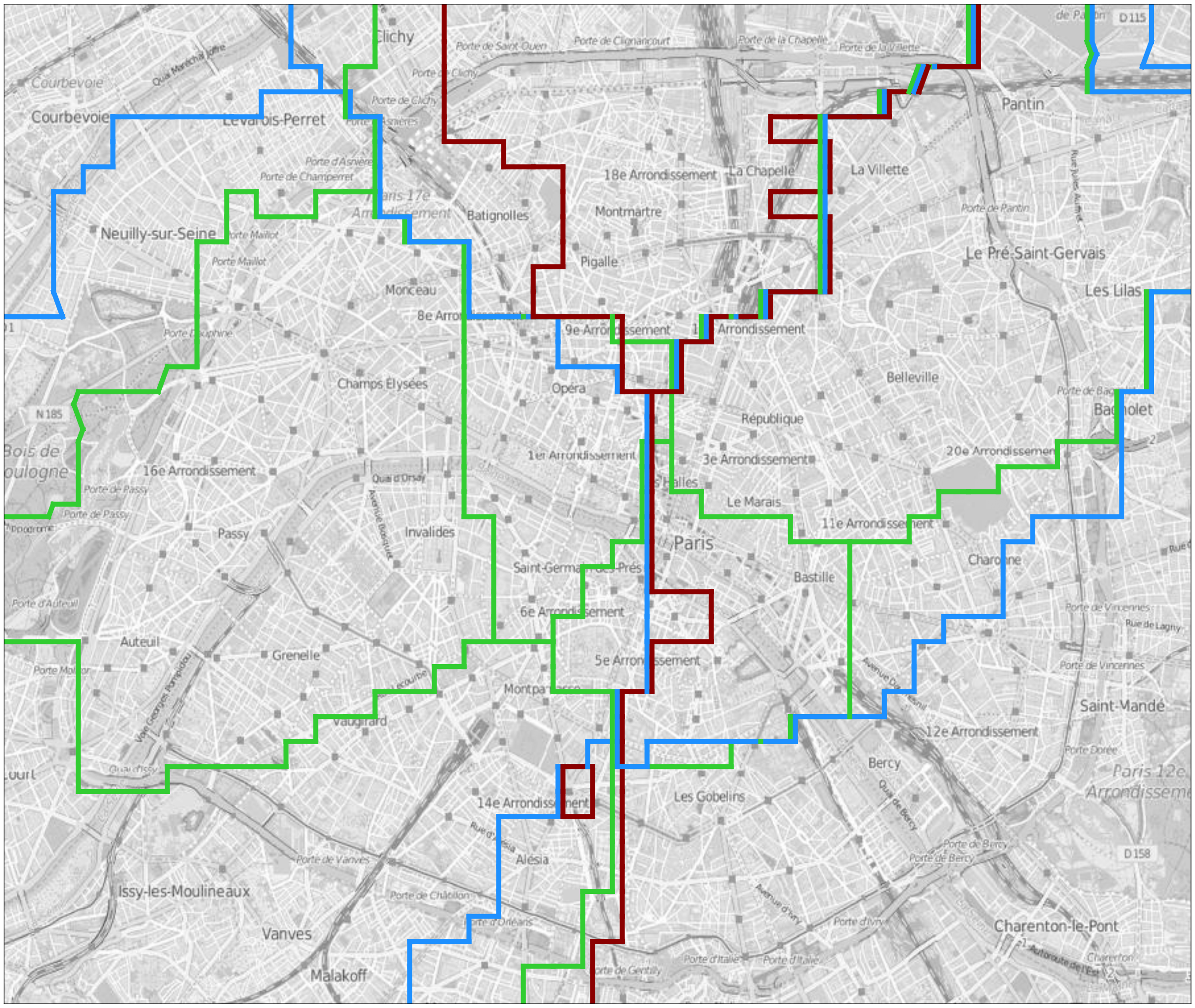

Figure S24: Paris, multi-scale borders. Map generated by the authors using the Basemap Matplotlib Toolkit ver. 1.0.8 (http://matplotlib.org/basemap/). Map tiles used in the background ©OpenStreetMap contributors, licensed under CC BY-SA (www.openstreetmap.org/copyright). The licence terms can be found on the following link: http://creativecommons.org/licenses/by-sa/2.0/ .

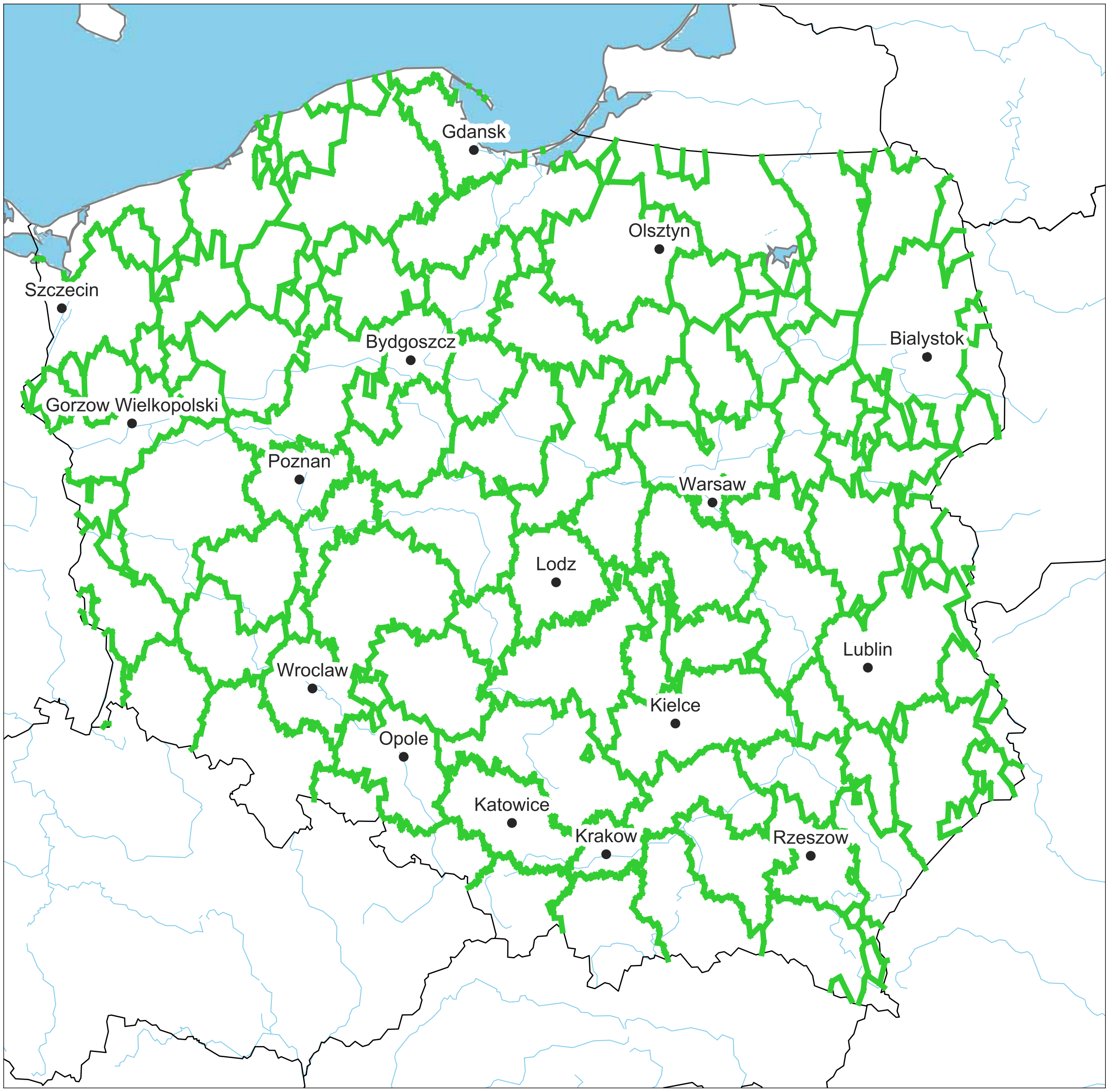

Figure S25: Poland, short-distance borders (percentile 20). Map generated by the authors using the Basemap Matplotlib Toolkit ver. 1.0.8 (http://matplotlib.org/basemap/).

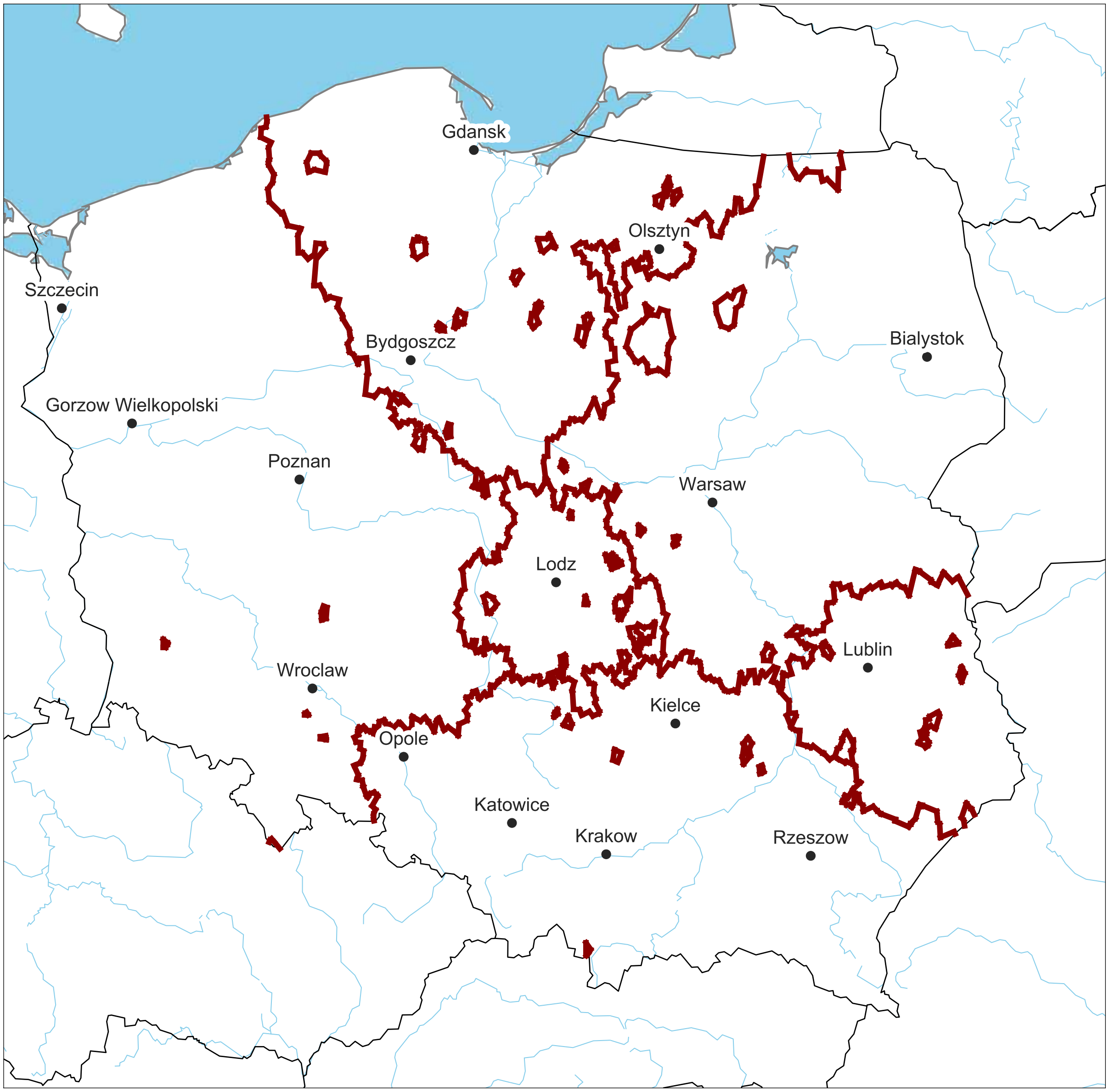

Figure S26: Poland, long-distance borders (percentile 79). Map generated by the authors using the Basemap Matplotlib Toolkit ver. 1.0.8 (http://matplotlib.org/basemap/).

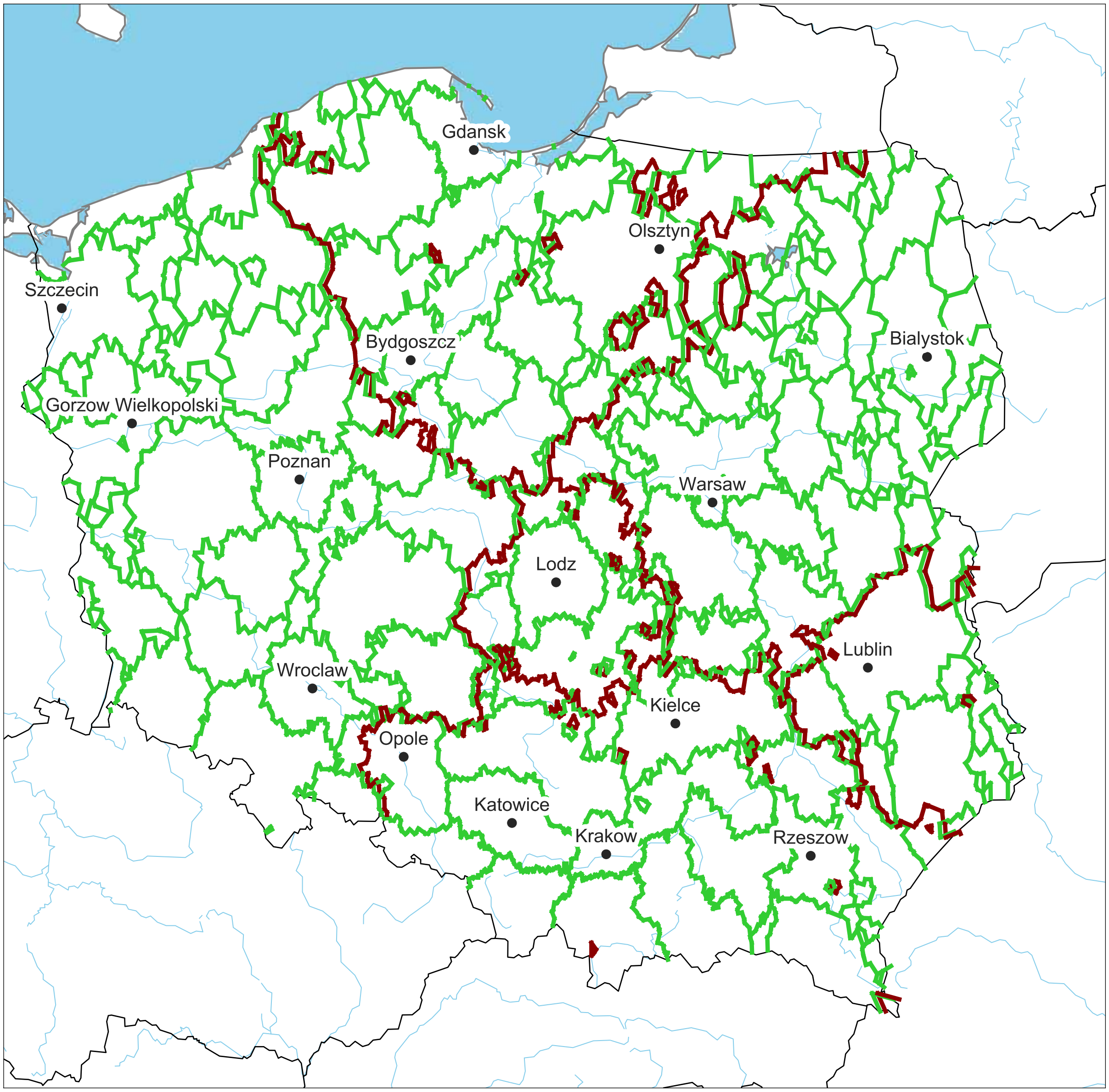

Figure S27: Poland, multi-scale borders. Map generated by the authors using the Basemap Matplotlib Toolkit ver. 1.0.8 (http://matplotlib.org/basemap/).

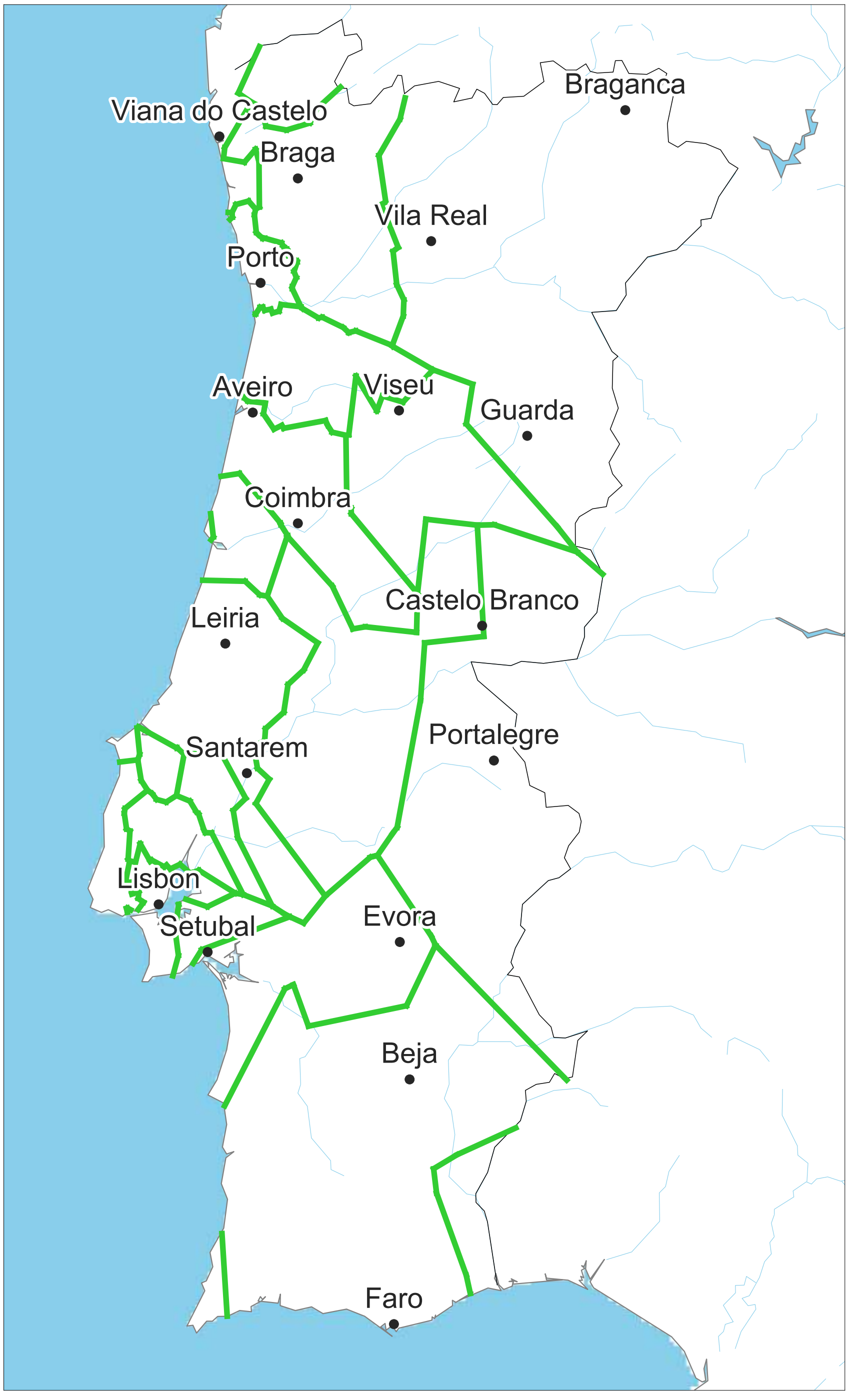

Figure S28: Portugal, short-distance borders (percentile 18). Map generated by the authors using the Basemap Matplotlib Toolkit ver. 1.0.8 (http://matplotlib.org/basemap/).

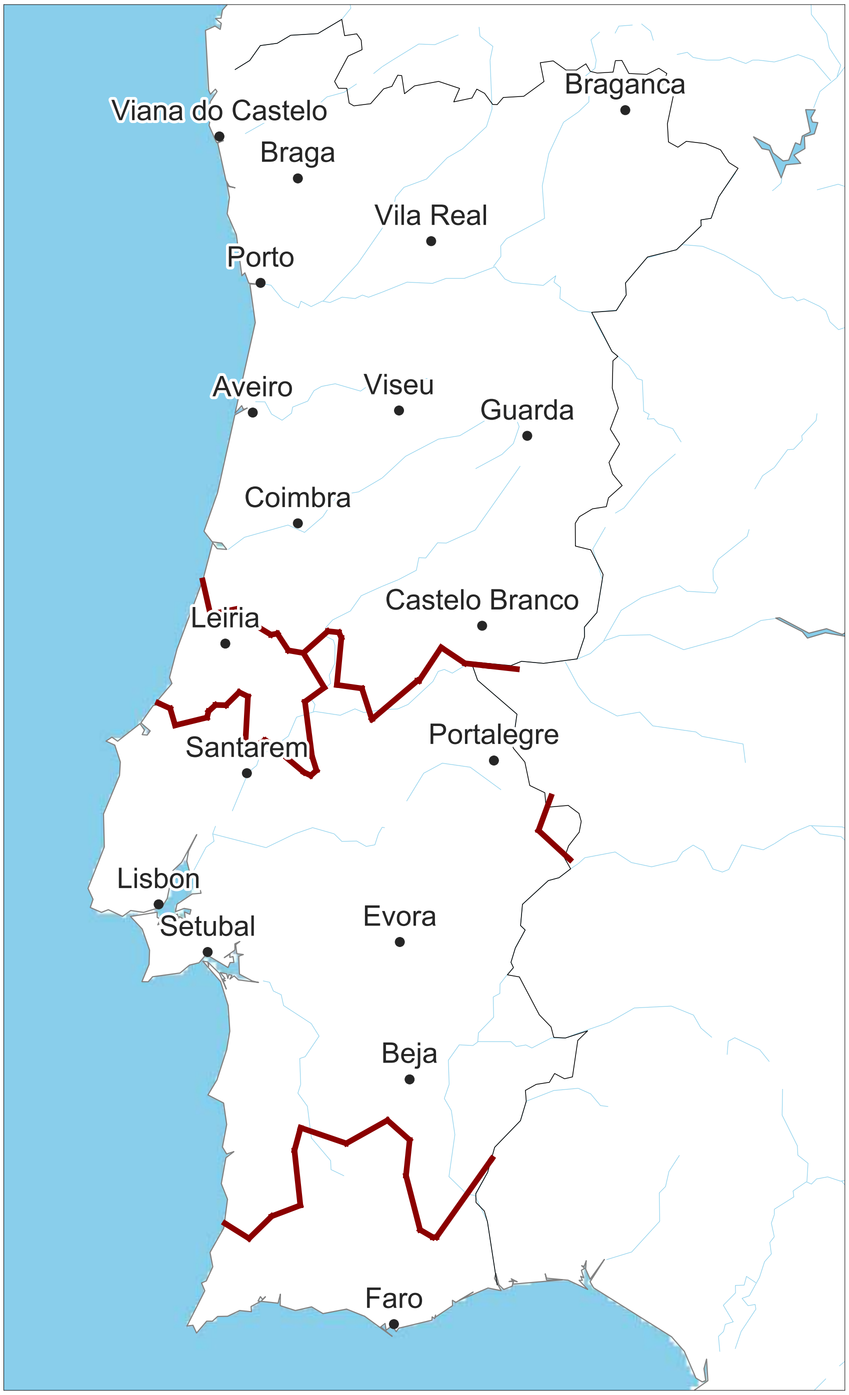

Figure S29: Portugal, long-distance borders (percentile 78). Map generated by the authors using the Basemap Matplotlib Toolkit ver. 1.0.8 (http://matplotlib.org/basemap/).

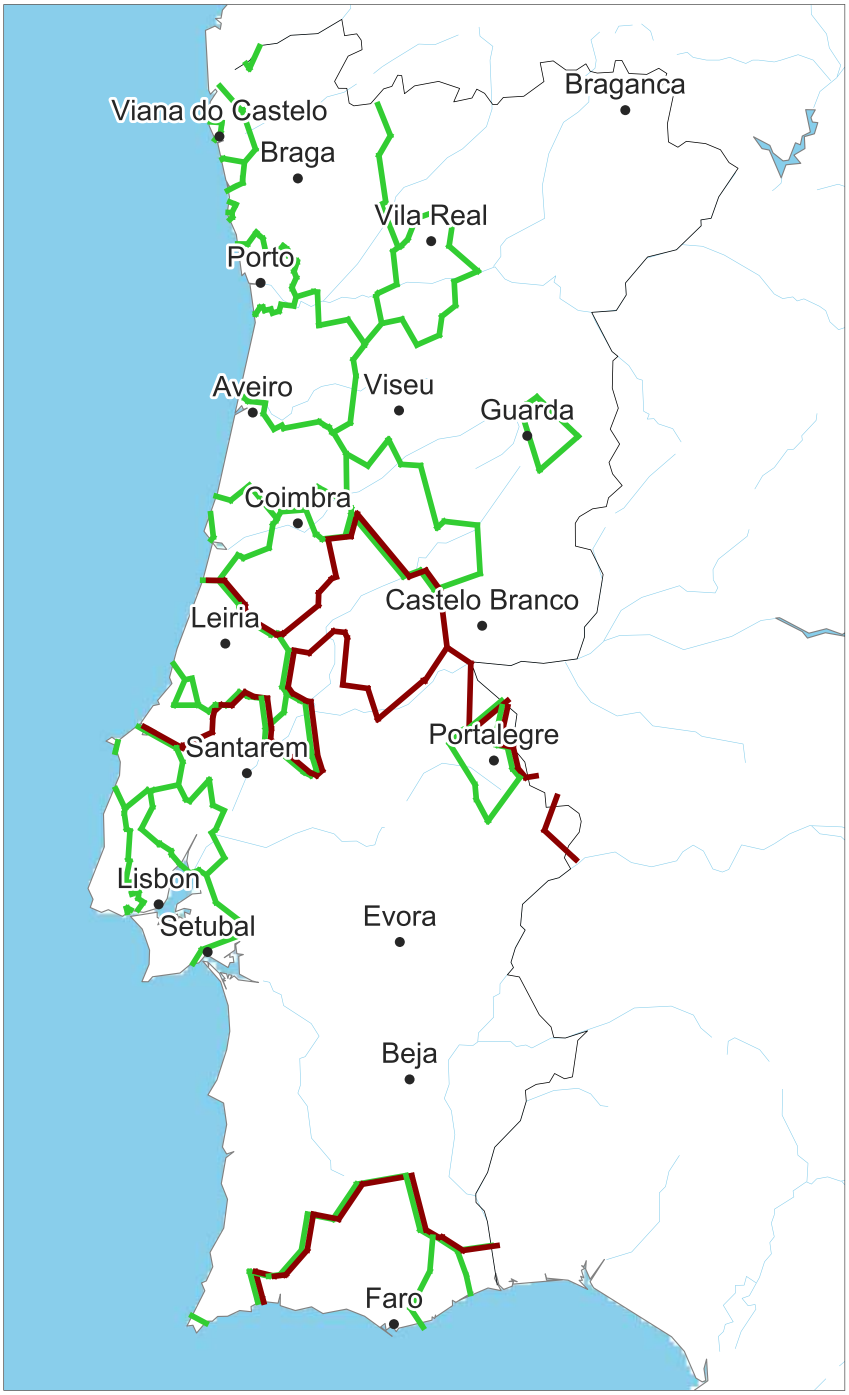

Figure S30: Portugal, multi-scale borders. Map generated by the authors using the Basemap Matplotlib Toolkit ver. 1.0.8 (http://matplotlib.org/basemap/).

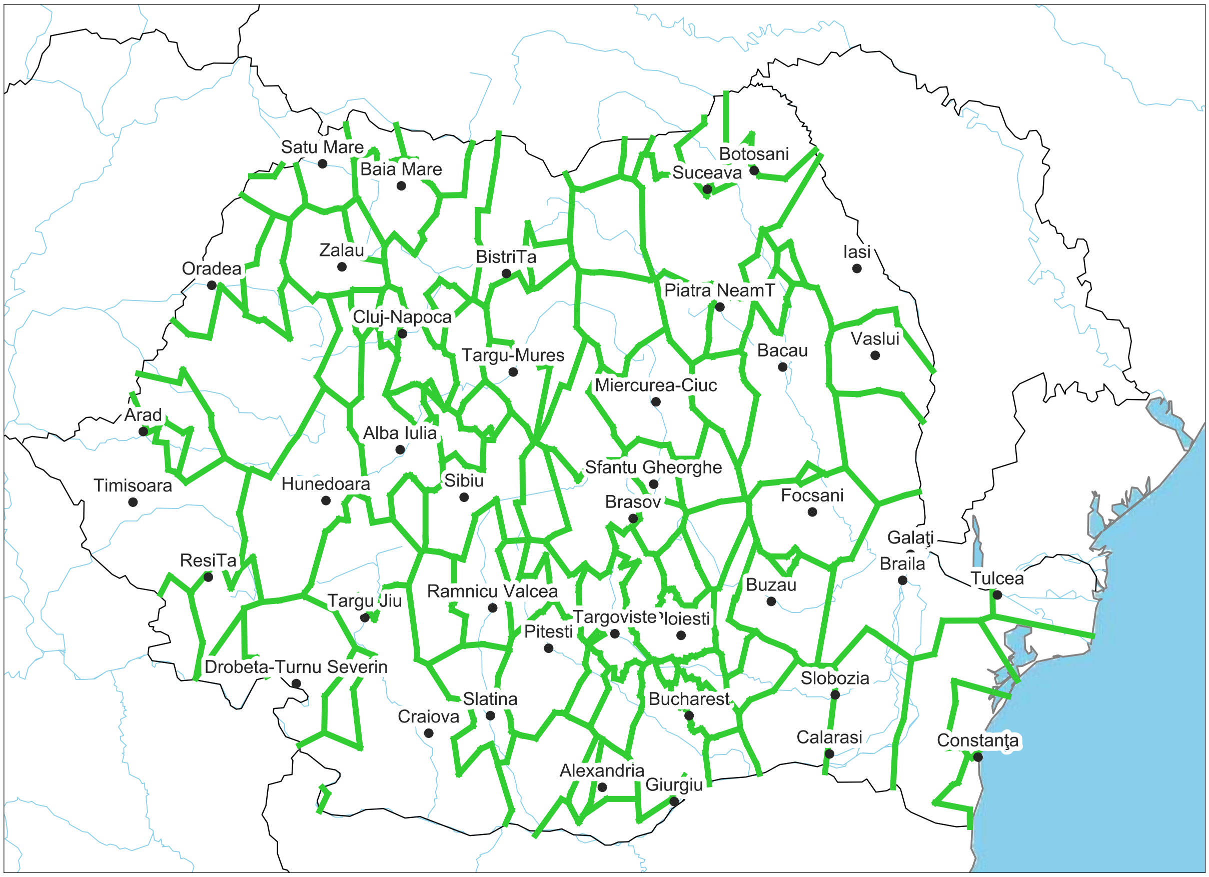

Figure S31: Romania, short-distance borders (percentile 8). Map generated by the authors using the Basemap Matplotlib Toolkit ver. 1.0.8 (http://matplotlib.org/basemap/).

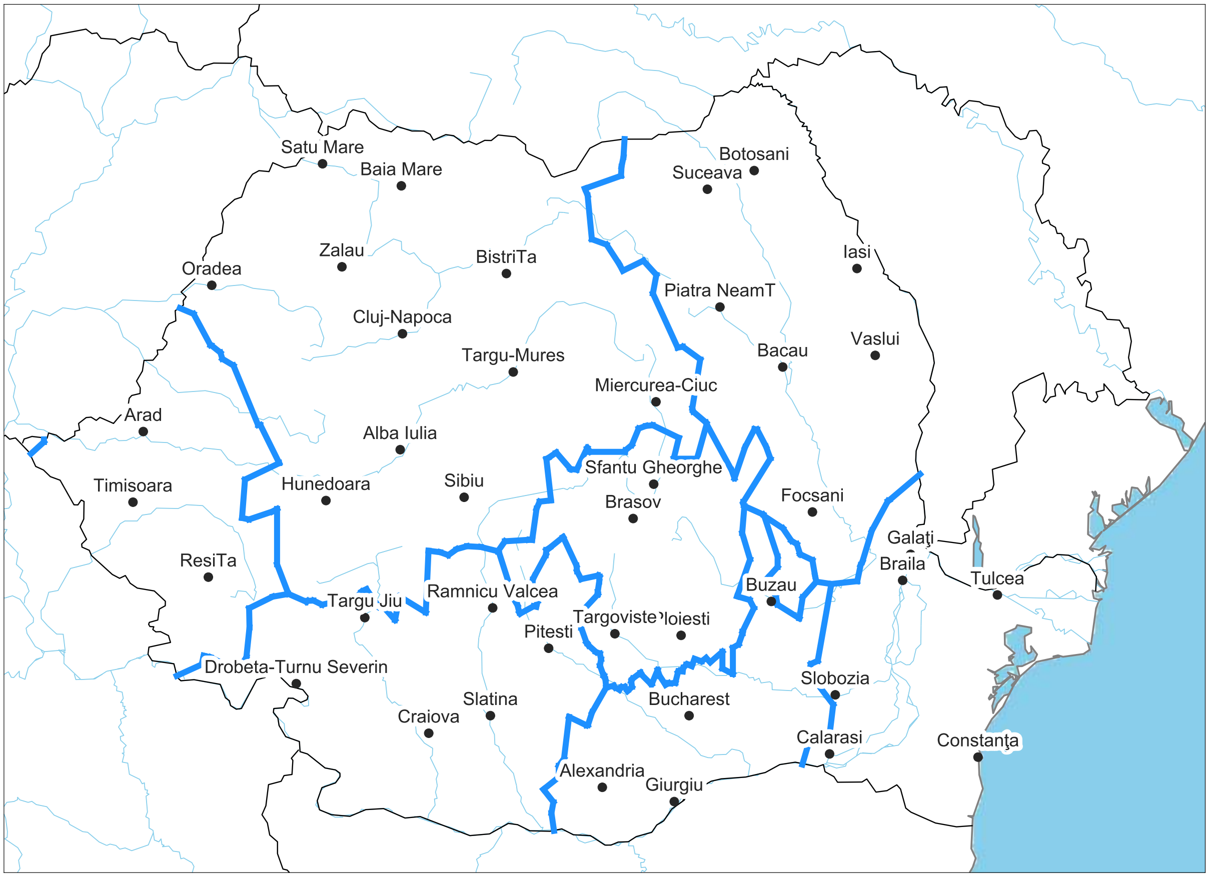

Figure S32: Romania, medium-distance borders (percentile 48). Map generated by the authors using the Basemap Matplotlib Toolkit ver. 1.0.8 (http://matplotlib.org/basemap/).

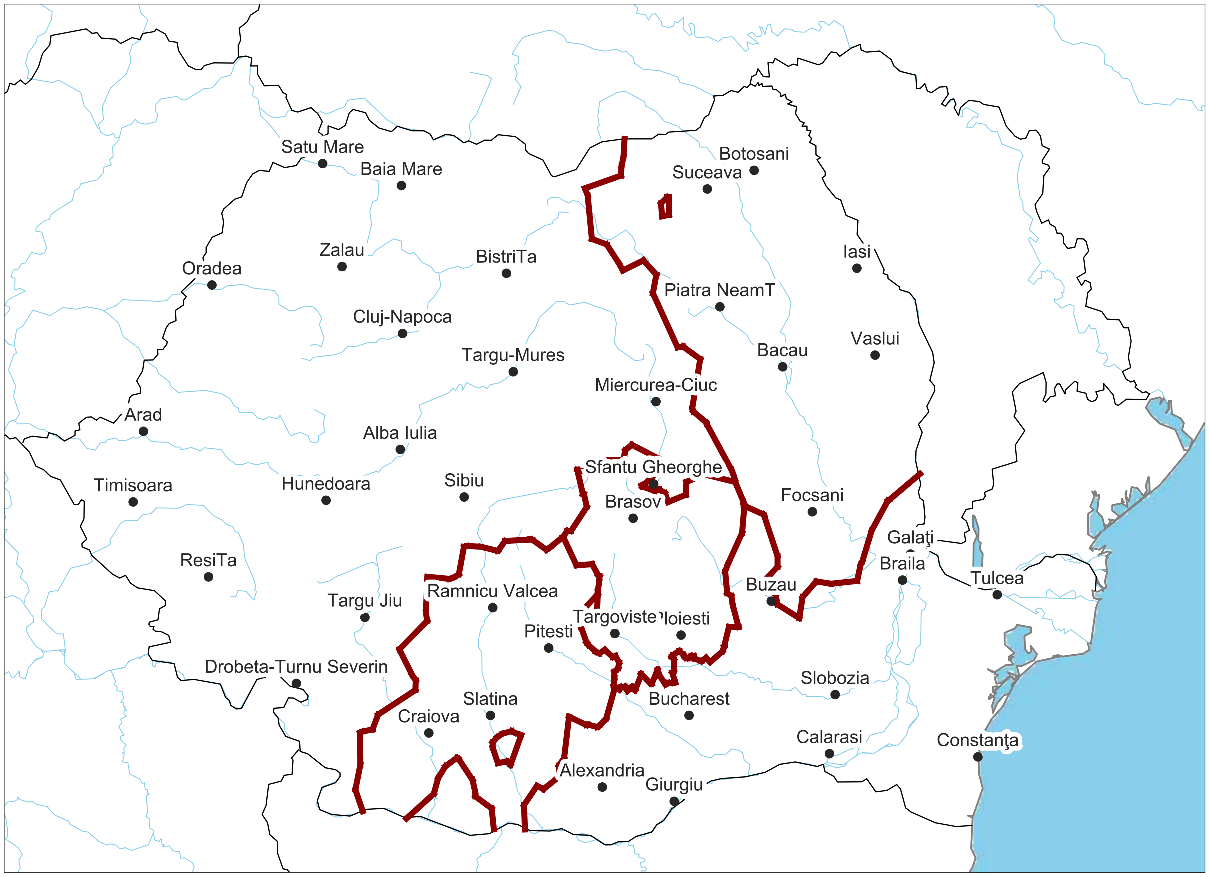

Figure S33: Romania, long-distance borders (percentile 77). Map generated by the authors using the Basemap Matplotlib Toolkit ver. 1.0.8 (http://matplotlib.org/basemap/).

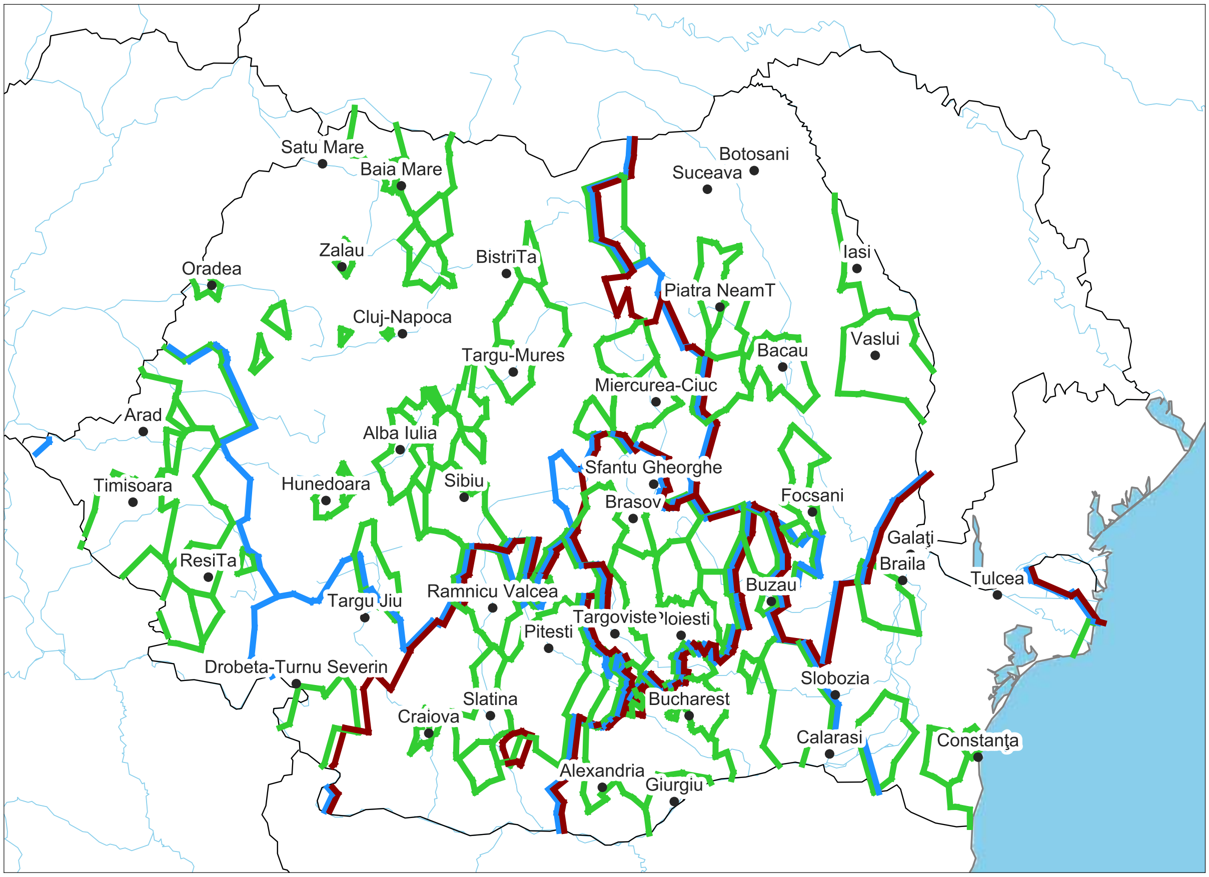

Figure S34: Romania, multi-scale borders. Map generated by the authors using the Basemap Matplotlib Toolkit ver. 1.0.8 (http://matplotlib.org/basemap/).

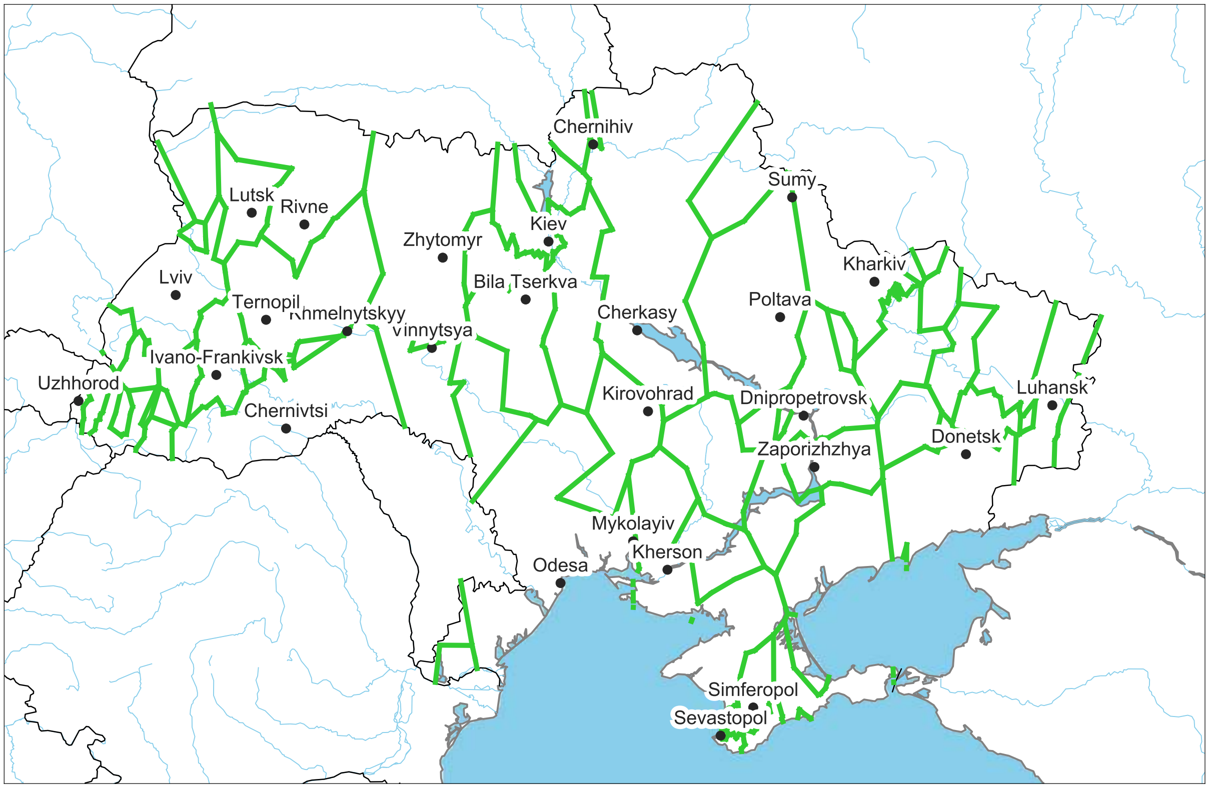

Figure S35: Ukraine, short-distance borders (percentile 32). Map generated by the authors using the Basemap Matplotlib Toolkit ver. 1.0.8 (http://matplotlib.org/basemap/).

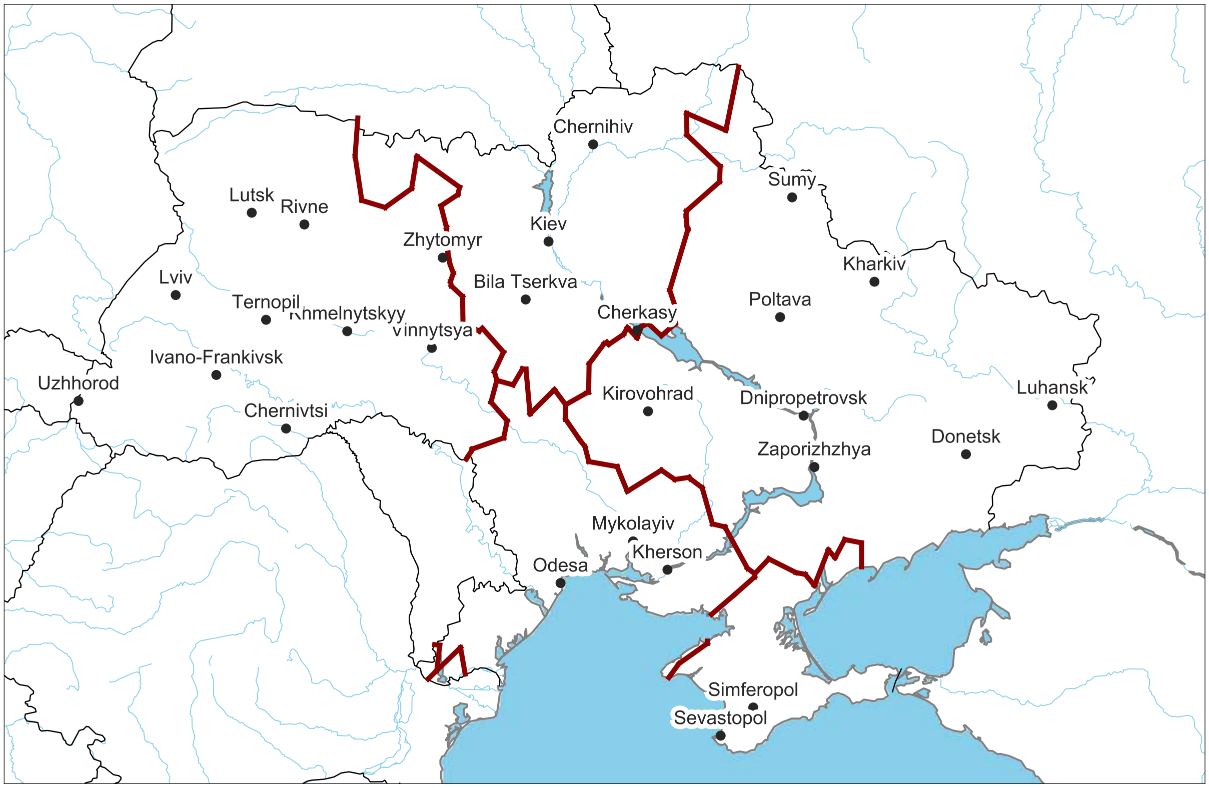

Figure S36: Ukraine, long-distance borders (percentile 80). Map generated by the authors using the Basemap Matplotlib Toolkit ver. 1.0.8 (http://matplotlib.org/basemap/).

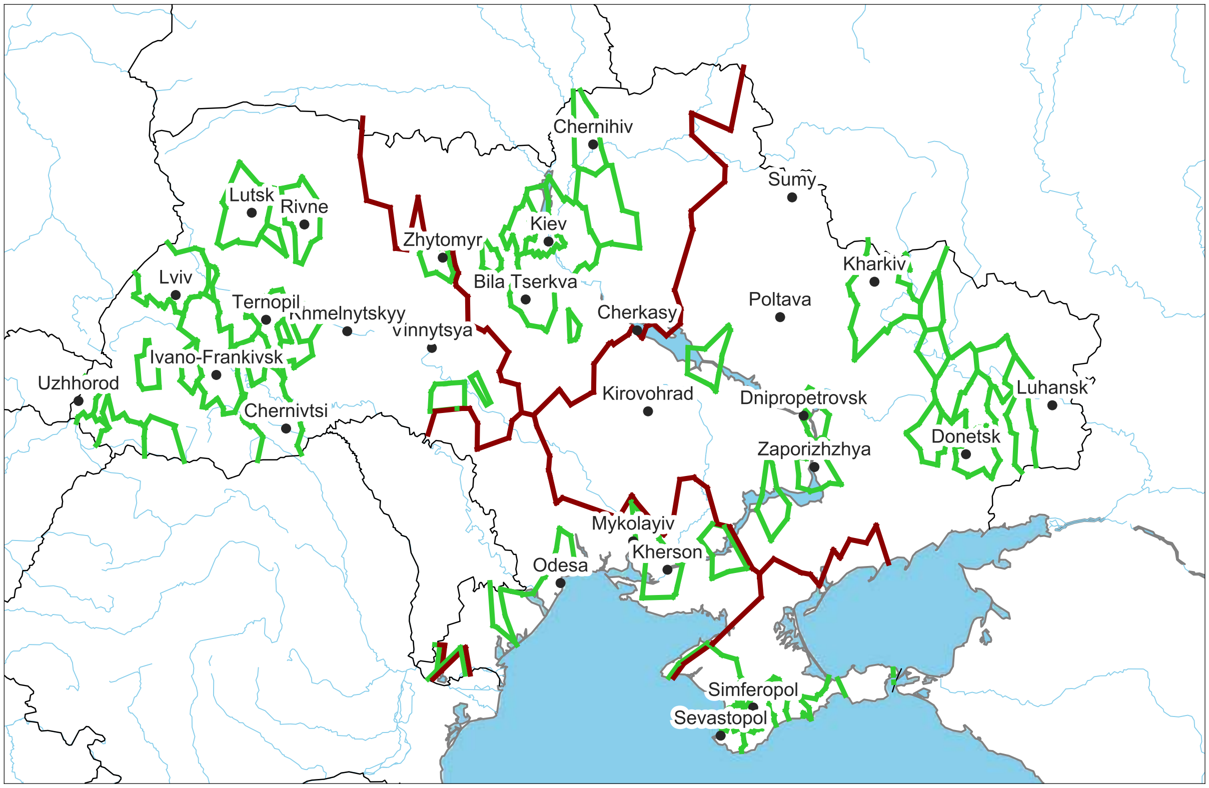

Figure S37: Ukraine, multi-scale borders. Map generated by the authors using the Basemap Matplotlib Toolkit ver. 1.0.8 (http://matplotlib.org/basemap/).
